# Genetic divergence and fine scale population structure of the common bottlenose dolphin (*Tursiops truncatus*, Montagu) found in the Gulf of Guayaquil, Ecuador

**DOI:** 10.7717/peerj.4589

**Published:** 2018-04-09

**Authors:** Rosa de los Ángeles Bayas-Rea, Fernando Félix, Rommel Montufar

**Affiliations:** 1 Escuela de Ciencias Biológicas, Pontificia Universidad Católica del Ecuador, Quito, Ecuador; 2 Museo de Ballenas, Salinas, Ecuador

**Keywords:** Genetic structure, *Tursiops truncatus*, Gulf of Guayaquil, Phylogeny, Phylogeography, Ecuador, mtDNA, Pacific Ocean

## Abstract

The common bottlenose dolphin, *Tursiops truncatus*, is widely distributed along the western coast of South America. In Ecuador, a resident population of bottlenose dolphins inhabits the inner estuarine area of the Gulf of Guayaquil located in the southwestern part of the country and is under threat from different human activities in the area. Only one genetic study on South American common bottlenose dolphins has been carried out to date, and understanding genetic variation of wildlife populations, especially species that are identified as threatened, is crucial for defining conservation units and developing appropriate conservation strategies. In order to evaluate the evolutionary link of this population, we assessed the phylogenetic relationships, phylogeographic patterns, and population structure using mitochondrial DNA (mtDNA). The sampling comprised: (i) 31 skin samples collected from free-ranging dolphins at three locations in the Gulf of Guayaquil inner estuary, (ii) 38 samples from stranded dolphins available at the collection of the “Museo de Ballenas de Salinas,” (iii) 549 mtDNA control region (mtDNA CR) sequences from GenBank, and (iv) 66 concatenated sequences from 7-mtDNA regions (*12S rRNA*, *16S rRNA*, NADH dehydrogenase subunit I–II, cytochrome oxidase I and II, cytochrome b, and CR) obtained from mitogenomes available in GenBank. Our analyses indicated population structure between both inner and outer estuary dolphin populations as well as with distinct populations of *T. truncatus* using mtDNA CR. Moreover, the inner estuary bottlenose dolphin (estuarine bottlenose dolphin) population exhibited lower levels of genetic diversity than the outer estuary dolphin population according to the mtDNA CR. Finally, the estuarine bottlenose dolphin population was genetically distinct from other *T. truncatus* populations based on mtDNA CR and 7-mtDNA regions. From these results, we suggest that the estuarine bottlenose dolphin population should be considered a distinct lineage. This dolphin population faces a variety of anthropogenic threats in this area; thus, we highlight its fragility and urge authorities to issue prompt management and conservation measures.

## Introduction

Identifying intraspecific variation of threatened coastal wildlife populations is essential for defining conservation units and developing appropriate conservation and management strategies. The evolutionary potential of species depends on genetic variation, which constitutes genetic diversity, differentiation, and distance (Frankham, Ballou & Briscoe, 2010; Taylor, Martien & Morin, 2010; Allendorf, Luikart & Aitke, 2013). Taken together, these factors contribute to defining evolutionary units as evolutionary significant units or management units (MUs) (Funk et al., 2012). However, understanding the intraspecific variation of species in animals like cetaceans, which inhabit broad geographical ranges with few or no geographical boundaries, is a challenge. Although cetaceans are highly mobile, many species have adapted to local conditions (Hoelzel, Potter & Best, 1998), leading to their local differentiation and subsequent evolution into new species. At the intraspecific level, the systematics of *Tursiops truncatus* has not been well-defined, suggesting different lineages in this species (Moura et al., 2013) due to habitat specialization (Caballero et al., 2012). In particular, it has been proposed that coastal populations are genetically well-differentiated from each other globally (Natoli, Peddemors & Hoelzel, 2004; Tezanos-Pinto et al., 2009).

The genus *Tursiops* includes two accepted species based on molecular and morphological differences: *T. truncatus* (Montagu, 1821) with global distribution and *T. aduncus* (Ehrenberg, 1833) restricted to the Pacific and eastern Indian Oceans. *T. truncatus*, the common bottlenose dolphin, has two recognized subspecies: *T. truncatus ponticus* in the Black Sea and *T. truncatus truncatus* in the Mediterranean Sea (Wang, Riehl & Dungan, 2014). Some populations have been proposed as a different species: the *aduncus*-type dolphin from South Africa, the western Pacific, and the eastern Indian Oceans (Natoli, Peddemors & Hoelzel, 2004; Perrin et al., 2007; Oremus et al., 2015); the Burrunan dolphin (“*Tursiops australis*”) from the Australian continent (Charlton, Taylor & McKechnie, 2006; Möller et al., 2008; Charlton-Robb et al., 2011; Moura et al., 2013); and *Tursiops gephyreus* from the South Atlantic (Carrion-Wickert et al., 2016), although its status as a separate species is not currently accepted (Wang, Riehl & Dungan, 2014).

The common bottlenose dolphin (hereafter bottlenose dolphin) is widely distributed in pelagic and coastal waters, including sounds, bays, and estuaries (Wang, Riehl & Dungan, 2014). The ecological adaptation of *T. truncatus* to different environmental conditions has generated two well-differentiated ecotypes (coastal and offshore) based on osteological and genetic data (Tezanos-Pinto et al., 2009; Perrin et al., 2011; Caballero et al., 2012; Louis et al., 2014a; Lowther-Thieleking et al., 2015). The coastal ecotype is found within 1–7.5 km from shore (Torres et al., 2003; Bearzi, Saylan & Hwang, 2009) while the offshore ecotype occurs less than 1 km from shore (Bearzi, Saylan & Hwang, 2009); however, it may vary according to the specific ocean basin. Both ecotypes can be considered in sympatry or parapatry in particular geographic regions (Torres et al., 2003; Bearzi, Saylan & Hwang, 2009; Caballero et al., 2012; Costa et al., 2016). The offshore ecotype is present in pelagic, coastal, and insular waters (Hoelzel, Potter & Best, 1998; Sanino et al., 2005; Tezanos-Pinto et al., 2009), whereas the coastal ecotype inhabits bays and coastal, estuarine, and continental areas (Sellas, Wells & Rosel, 2005; Parsons et al., 2006). Additionally, the offshore ecotype is highly dispersed, has high levels of genetic diversity and lacks population structure (Hoelzel, Potter & Best, 1998; Natoli, Peddemors & Hoelzel, 2004; Quérouil et al., 2007). Conversely, most coastal populations show population structure at small geographic scales with low genetic diversity at the regional scale (Rosel, Hansen & Hohn, 2009; Mirimin et al., 2011; Fruet et al., 2014).

Although the bottlenose dolphin is widely present along the entirety of the western coast of South America (Wang, Riehl & Dungan, 2014), only one genetic study has been carried out to date. Sanino et al. (2005) evaluated the genetic diversity and the phylogenetic relationships of two offshore groups and two coastal groups from Peru and Chile. Among these populations, three different populations (one offshore Peruvian–Chilean population and two different coastal Peruvian and Chilean populations) were found. In Ecuador, information on the bottlenose dolphin is scarce, but the presence of coastal and offshore ecotypes in Ecuadorian waters has been proposed in previous studies (Félix, 1997; Félix et al., 2017a; Jiménez & Alava, 2014; Palacios, Salazar & Day, 2004). The coastal ecotype, with a sub-structured population in semi-closed communities (Félix, 1997; Félix et al., 2017a), is found in the Gulf of Guayaquil inner estuary, including the Jambelí Channel (Félix, 1994, 1997; Félix et al., 2017a), Estero Salado (Félix et al., 2017a), Posorja, and El Morro and Bajo Alto mangroves (Jiménez & Alava, 2014; Félix et al., 2017a), while the offshore ecotype is observed around the Galápagos Islands and in pelagic waters (Palacios, Salazar & Day, 2004). Although the bottlenose dolphin in the inner estuary is recognized as a coastal ecotype, no information about its genetic identity is available.

In the Gulf of Guayaquil inner estuary, two photo identification studies were carried out in the 1990s focusing on the behavioral ecology, social organization, and social structure of the bottlenose dolphin population (Félix, 1994, 1997). The social organization of this population was characterized as a hierarchically structured society in which females are organized in bands while males form alliances to obtain dominant status and access mature females (Félix, 1997). In addition, dolphins form small groups ranging from two to eight individuals and pods ranging from 10 to 25 individuals so they can forage at a specific site and strand fish on the shore (Jiménez & Alava, 2014). To date, there is no actual estimate of dolphin population size in the whole Gulf of Guayaquil. However, more recent studies described two communities: one community of around 70 dolphins in the Estero Salado (Félix et al., 2017a) and another single resident community of around 43–45 dolphins inhabiting Morro Channel within the Morro Mangrove Wildlife Refuge (2°39′S and 80°11′W) (Jiménez & Alava, 2014; Félix et al., 2017a), a protected area of approximately 10,130 ha of mangrove forest (Ministerio del Ambiente del Ecuador (MAE), 2010).

The bottlenose dolphins in the Gulf of Guayaquil inner estuary reside in an area characterized by high biological productivity. During the cold, dry season, the Humboldt Current and superficial subtropical water mass promote outcrops of high-nutrient waters with increased biological productivity (Twilley et al., 2001). There are approximately 122,437 ha of mangrove forest in the estuary; nevertheless, it is one of the most fragile and threatened ecosystems in the country (MAE, 2010). This ecosystem is located near Guayaquil, the country’s largest and most populated city and one of its four major ports (Montaño & Sanfeliu, 2008; Castro et al., 2012). Approximately 45% of Ecuador’s population lives around the inner estuary, which is its most important production area for industrial and artisanal fisheries (Montaño & Sanfeliu, 2008).

The bottlenose dolphin population of the Gulf of Guayaquil inner estuary (hereafter referred to as the estuarine bottlenose dolphin) is one of the most vulnerable populations due to human population increase and coastal development activities (Jiménez et al., 2011; Jiménez & Alava, 2014; Félix et al., 2017b). This has resulted in a significant population reduction in the last decades (Jiménez & Alava, 2014; Félix et al., 2017a). The main threats affecting the species in the estuary are collisions between vessels and dolphins (Van Waerebeek et al., 2007; Félix et al., 2017b), bycatch (Van Waerebeek et al., 1997; Félix et al., 2017b), ship-based tourism that includes mismanaged dolphin tourism carried out by locals (Félix, 1997; Félix et al., 2017a), habitat degradation, dredging activities, and intense fishing (Jiménez & Alava, 2014). Furthermore, the presence of biological and chemical contaminants in both the sediment and the water column (Castro et al., 2012) can impact dolphin health, generating lobomycosis-like or lacaziosis-like infectious diseases (Van Bressem et al., 2015). Due to its vulnerability, this species is considered Vulnerable (VU) according to the Ecuadorian Mammal Red List and is protected by Ecuadorian law, meaning hunting and trade are prohibited in the country indefinitely (Jiménez et al., 2011). Despite the species being protected, the dolphin population is facing a variety of hazards that could, directly and indirectly, affect its survival and long-term adaptability.

The aim of this study is to assess the evolutionary link of the estuarine bottlenose dolphin in the Gulf of Guayaquil with *T. truncatus* populations elsewhere based on analyses of several mitochondrial DNA (mtDNA) regions. The genetic study was conducted using: (i) a 392 bp fragment of mtDNA control region (mtDNA CR) to estimate the genetic diversity of the bottlenose dolphin of the Gulf of Guayaquil; (ii) 397 bp mtDNA CR sequences to assess population divergence, phylogeographic patterns, and phylogeny between the estuarine bottlenose dolphins and bottlenose dolphin populations elsewhere; and (iii) 7-mtDNA regions (5,209 bp) to determine phylogenetic relationships including distinct *T. truncatus* populations and all species inside the genus *Tursiops*. To our knowledge, this is the first genetic study carried out with bottlenose dolphins in the Gulf of Guayaquil. The results will be discussed in terms of conservation. The estuarine bottlenose dolphin is Ecuador’s most vulnerable dolphin population because of anthropogenic threats, so understanding population boundaries is essential to improving local management strategies and ensuring the short-term conservation of the bottlenose dolphin in the area.

## Materials and Methods

### Study area

**Figure 1:**
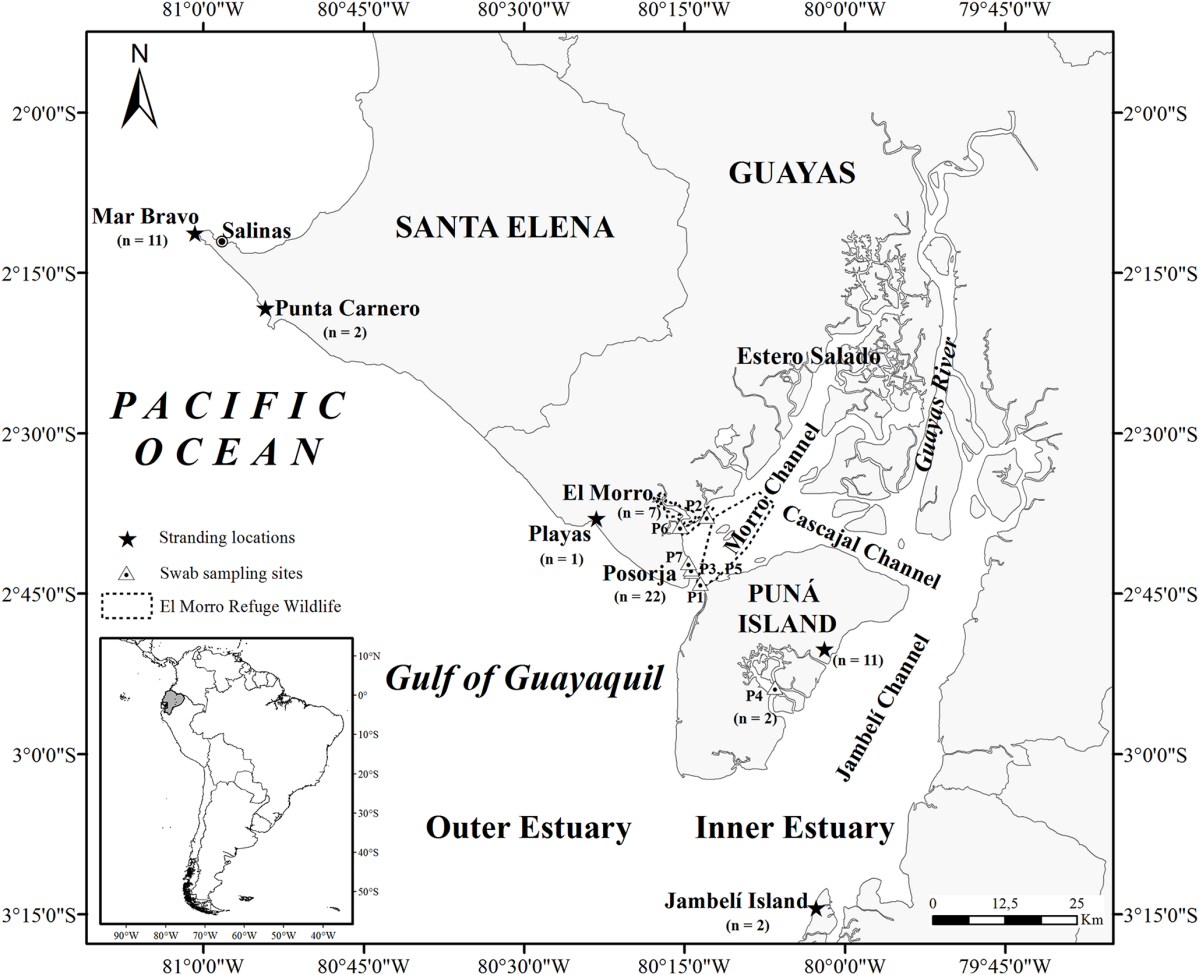
Map of the Gulf of Guayaquil showing sample locations. Stranded samples were obtained in four locations in the Gulf of Guayaquil. Mar Bravo (Ttr_3–Ttr_8, Ttr_32, Ttr_34-Ttr_37), Punta Carnero (Ttr_2, Ttr_18), Playas (Ttr_17), Jambelí Island (Ttr_10, Ttr_33), and east side of Puná Island (Ttr_9, Ttr_11, Ttr_12, Ttr_15, Ttr_16, Ttr_19-Ttr_21, Ttr_23, Ttr_25, Ttr_31). The sampling sites in the inner estuary are represented by the letter P, which indicates the GPS points where skin sampling of free-ranging dolphins took place. Posorja (*n* = 22; P1: Ttr_38-Ttr_46, P3: Ttr_49-Ttr_52, P5: Ttr_55-Ttr_57, P7: Ttr_63-Ttr_68), El Morro (*n* = 7; P2: Ttr_47-Ttr_48, P6: Ttr_58-Ttr_62), and the east side of Puná Island (*n* = 2; P4: Ttr_53, Ttr_54).

The Gulf of Guayaquil, the largest estuary on the southeast Pacific coast, is located in southwestern Ecuador (3°S and 81°W) (Fig. 1) (Stevenson, 1981; Montaño & Sanfeliu, 2008). The entrance to the gulf is 200 km wide, stretching from Santa Elena (2°12′S, Ecuador) to near Máncora (4°07′S, Peru) and extending 130 km inland. The Gulf of Guayaquil includes an outer and inner estuary (Twilley et al., 2001) divided by the western part of Puná Island. The outer estuary begins on the western side of Puná Island (80°15′W) and ends at 81°W. The inner estuary extends inland in several directions, surrounding a complex of islands covered in mangroves and channels. The inner estuary includes most of Puná Island, which lies between three channels: the Morro Channel, stretching 3 km northeast; the Cascajal Channel in the north; and the Jambelí Channel, extending 11–28 km wide to the east. In the northern part of Puná Island, the Cascajal Channel connects the Estero Salado to the Guayas River (Stevenson, 1981) while in the east; the Jambelí Channel connects the outer estuary with the Guayas River.

The weather in the study area is characterized by two seasons: one warm, rainy from January to May, and the cold, dry from June to November (Stevenson, 1981). Rainfall is seasonal, and more than 95% of precipitation occurs from December to May, causing seasonal river discharge (Stevenson, 1981; Twilley et al., 2001). More than 20 hydrographic basins contribute to a large drainage area in the Gulf of Guayaquil (Twilley et al., 2001; Montaño & Sanfeliu, 2008), of which the Guayas River Basin is the main contributor of freshwater to the estuary (Stevenson, 1981). In the outer estuary, the surface water temperature varies from 21.5 °C to 25 °C during the dry and rainy seasons, respectively. In the inner estuary, water surface temperature fluctuates between 25 °C during the dry season and 28 °C during the rainy season (Stevenson, 1981). Finally, the tides vary from 1.8 m near the upper boundary of the Gulf to 3–5 m near the city of Guayaquil (Twilley et al., 2001).

### Sample collection

We included 38 museum samples, which are available from the “Museo de Ballenas de Salinas” (Salinas Whale Museum) collection in the Salinas city. The samples belong to the mentioned museum according to the patent of wildlife management granted by the Department of Salinas, Ministry of Environment of Ecuador (patent number FAU-0002-DPSE/VS). Of 38 museum samples, one was a skin sample (Ttr_1) from a Galápagos free-ranging dolphin, and 37 (Ttr_2–Ttr_37 and Ttr_69) were obtained from stranded dolphins (eight skin and 29 bone samples). Stranded samples were collected from different years (1990–1996, 2001, 2005–2010, and 2013) and locations, including the outer (Mar Bravo and Punta Carnero seashores located in Salinas, and Playas; *n* = 14) and inner (eastern part of Puná Island and Jambelí Island; *n* = 13) estuary of the Gulf of Guayaquil, Galápagos Islands (*n* = 1), and Santa Rosa, Peru (*n* = 1). The other stranded samples (*n* = 8) were collected in different undefined locations from the Gulf of Guayaquil. Occipital condyle and mandible bone powder were gathered based on the technique reported by Morin et al. (2006). Museum sample information is summarized in Table S1.

Additionally, we collected 31 skin samples (Ttr_38–Ttr_68) from free-ranging dolphins at three different sites (Posorja: *n* = 22, El Morro: *n* = 7, and east side of Puná Island: *n* = 2) from the Gulf of Guayaquil inner estuary (Fig. 1). The field work took place between March and August 2013. We took swab samples from the dorsal-lateral region of the dolphins based on the non-invasive technique reported by Harlin et al. (1999) with a few modifications: a sterilized square piece of sand paper 5 × 5 cm was used instead of a nylon scrub pad wrapped around the tip of a pole. Sample information is summarized in Table S1. Skin samples were removed with sterilized forceps and stored in 100% ethanol for subsequent genetic analyses. Free-ranging dolphin samples were collected following the guidelines specified by the research permits given by the Department of Guayas Province, Ministry of Environment of Ecuador (permit number 004-IC-FAU-DPG/MAE). No ethical approval was considered necessary and the sampling technique was not submitted for ethical analysis because the animals were not handled directly and a non-invasive sampling technique was used. We performed all genetic analyses in the Ecology and Genetics Laboratory at the Pontificia Universidad Católica del Ecuador (Pontifical Catholic University of Ecuador).

### DNA extraction

Total genomic DNA from 40 skin samples (31 free-ranging dolphin samples and nine museum samples) was isolated using a modified proteinase K digestion protocol and two chloroform: isoamyl alcohol (24:1) extractions followed by ethanol precipitation (Green & Sambrook, 2012). We extracted DNA from the bone powder of 29 samples using a Wizard Genomic DNA Purification Kit (Promega, Madison, WI, USA), following the manufacturer’s protocol. DNA from bone samples were extracted in a dolphin DNA-free laboratory, and DNA manipulation was carried out based on those procedures described by Morin et al. (2006). The concentration and purity of genomic DNA were analyzed using a NanoDrop spectrophotometer (ThermoFisher Scientific, Waltham, MA, USA).

### Molecular sex determination

We identified the sex of all free-ranging and stranded dolphins (*n* = 69) with a duplex polymerase chain reaction (PCR) amplification using two sets of primers, ZFX0582F and ZFX0923R (Bérubé & Palsbøll, 1996), to target a partial fragment of the *ZFX* gene, as well as PMSRYF (Richard, McCarrey & Wright, 1994) and TtSRYR (Rosel, 2003) to amplify a partial fragment of the *SRY* gene. PCR amplification reactions were carried out based on those reported by Rosel (2003), with a few modifications. PCR amplification reactions were carried out in a 20 μl reaction mixture containing 20–30 ng of DNA, 1X PCR buffer, 1.5 mM MgCl_2_, 0.3 μM of each primer, 150 μM dNTPs, and 1.0 U of Taq DNA polymerase (Promega, Madison, WI, USA). PCR thermo-cycling conditions consisted of an initial denaturation step at 95 °C for 5 min, followed by 35 cycles of denaturation at 94 °C for 30 s, annealing at 51 °C for 45 s, and extension at 72 °C for 45 s with a final extension step at 72 °C for 10 min. Gender was determined by the banding pattern on a 2.5% agarose gel, stained with ethidium bromide, and visualized under ultraviolet light.

### mtDNA sequencing

For all samples (*n* = 69), we amplified 7-mtDNA regions (*12S rRNA*, *16S rRNA*, NADH dehydrogenase subunit I–II (*ND1-ND2*), cytochrome oxidase I and II, (*COI* and *COII*), cytochrome b (*Cyt b*), and the mtDNA CR) designed on the basis of the *T. truncatus* mitochondrial genome. The details of each set of primers are listed in Table S2. We chose the hypervariable region mtDNA CR (∼500 bp) for population structure and phylogeographic analyses for several reasons: (i) its fast mutation rate (Taylor, Martien & Morin, 2010), (ii) the availability of previously published sequences for this species in other parts of their range, and (iii) the small size of the PCR product is ideal for amplifying museum DNA samples. For the phylogenetic analyses, we included the 7-mtDNA regions to obtain a high phylogeny resolution due to the many informative sites of those regions.

Polymerase chain reaction amplification reactions were carried out in a 25 μl reaction mixture containing 20–30 ng of DNA, 1X PCR buffer, 1.5–2.0 mM MgCl_2_ (specific to each primer and detailed in Table S2), 0.4 μM of each primer, 200 μM dNTPs, and 1.0 U of Taq DNA polymerase (Promega, Madison, WI, USA). PCR thermo-cycling conditions consisted of 94 °C for 2 min, followed by 35 cycles of 94 °C for 30 s, annealing temperature (specific to each primer and detailed in Table S2) for 60 s, and extension at 72 °C for 60 s with a final extension step at 72 °C for 10 min. The PCR products were electrophoresed on a 1–1.5% agarose gel containing ethidium bromide, and visualized under ultraviolet light. All PCR products were purified for sequencing using exonuclease I and shrimp alkaline phosphatase (ExoSap-IT®). Both strands were sequenced by MACROGEN (Seoul, South Korea). All sequences were manually assembled and edited using BioEdit 7.2.3 software (Hall, 1999). In order to validate the data, all consensus sequences obtained were analyzed using the BLAST algorithm in GenBank (Altschul et al., 1997). Multiple alignments were performed for each gene using CLUSTAL W implemented in MEGA 6 software (Tamura et al., 2013) and each alignment was edited and checked visually. If we found unique sites, we rechecked the electropherogram alignments in both directions. All nucleotide sequences found in this study were deposited in GenBank under accession numbers KU991990–KU992299. The samples used in each test are summarized in Table S1.

### Genetic diversity

We used mtDNA CR from 48 free-ranging and stranded dolphins from the Gulf of Guayaquil to investigate genetic variability within and among sampling sites. We compared the variability of the 392 bp mtDNA CR between sequences obtained in the present study with a sequence of *T. truncatus* from the Gulf of California (accession number KF570389.1). This sequence was chosen because it represents the coastal ecotype mitogenome from the eastern Pacific Ocean (Moura et al., 2013). Unique haplotypes were detected using DNAsp v.5.10.01 (Librado & Rozas, 2009). We estimated genetic diversity by calculating the number of haplotypes using DNAsp v.5.10.01 (Librado & Rozas, 2009), haplotype diversity (*h*), and nucleotide diversity (π) with Arlequin v.3.5 (Excoffier & Lischer, 2010), as well as Tajima’s D test (Tajima, 1989) and Fu’s Fs test (Fu, 1997) of selective neutrality using Arlequin v3.5. We inferred the significance of both neutrality tests by randomization (10,000 steps).

### Population structure analyses

On a smaller spatial scale, we calculated the population genetic differentiation of 48 free-ranging and stranded dolphins from the Gulf of Guayaquil using 397 bp mtDNA CR truncated alignment. We evaluated the differentiation between the inner estuary dolphins, constituting four communities (Posorja: *n* = 22, El Morro: *n* = 7, Puná Island: *n* = 6, Jambelí Island: *n* = 2), and the outer estuary dolphins, represented by three communities (Mar Bravo: *n* = 9, Playas: *n* = 1, Punta Carnero: *n* = 1). On a broad geographic scale, population genetic differentiation was evaluated in both the inner and outer estuary dolphin populations from the Gulf of Guayaquil in addition to global populations. In the analyses, we incorporated 432 mtDNA CR sequences from different geographic regions made available in the GenBank. We implemented an analysis of molecular variance (AMOVA) to compute significance with 10,000 permutations (Excoffier, Smouse & Quattro, 1992). We used AMOVA based on haplotype data (*F*_ST_), haplotype frequency and genetic distance (φ_ST_) via Arlequin v.3.5 (Excoffier & Lischer, 2010) to estimate population genetic differentiation. For φ_ST_, we inferred the best-fitting model for nucleotide evolution under the Akaike information criterion (AIC) using jModelTest v.2.1.4 software (Darriba et al., 2012). The HKY+ I model was selected by jModelTest; however, we used the Tamura–Nei model (Tamura & Nei, 1993), as it is the closest model to the HKY+ I. We tested *F*_ST_ and φ_ST_ significance levels with 10,000 permutations. We estimated pairwise genetic differentiation of *F*_ST_ and φ_ST_ between both populations with 10,000 permutations.

### Population divergence analyses

A total of 549 published mtDNA CR sequences were included in the analyses with 50 sequences of this study to compare dolphin populations from the Gulf of Guayaquil with global populations (accession numbers in Table S3). We excluded populations with less than five available sequences from the test of divergence. We aligned all sequences using CLUSTAL W implemented in MEGA 6 software (Tamura et al., 2013), and each alignment was edited and checked visually. Given the sequences were different lengths, the analyses were based upon 397, 360, and 317 bp truncated alignment. We estimated the average evolutionary divergence between (*d_XY_*) different geographic locations and the number of net nucleotide substitutions per site between populations (*d_A_*) using Tamura–Nei model via MEGA 6 software (Tamura et al., 2013). The estimator *d_A_* calculated the differentiation between populations and subspecies (Rosel et al., 2017).

### mtDNA CR phylogeographic analyses

We incorporated 374 mtDNA CR sequences from different geographic regions made available in the GenBank database with 53 sequences of this study to investigate the phylogeographic relationships among haplotypes located in the Gulf of Guayaquil and other populations of *T. truncatus* elsewhere (accession numbers in Table S3). We aligned all sequences using CLUSTAL W implemented in MEGA 6 software (Tamura et al., 2013). Given the sequences were different lengths, the analysis was based on 397 bp truncated alignment. The genealogical relationships were inferred using median-joining network implemented in Network v.4.6.0 software (Bandelt, Forster & Röhl, 1999). The consensus sequence is detailed in Supplemental Information 1.

### Phylogenetic analyses

We constructed two phylogenetic trees to infer the phylogenetic relationships of the Gulf of Guayaquil bottlenose dolphins based on Bayesian inference using MrBayes v.3.2.2 (Ronquist et al., 2012). A single tree was constructed with 257 published mtDNA CR haplotypes and 14 haplotypes obtained in this study. One outgroup was the rough-toothed dolphin *Steno bredanensis* mtDNA CR sequence (accession number JF339982.1). The details of mtDNA CR haplotypes and their corresponding GenBank accession number are summarized in Table S4. We inferred the best-fitting model for nucleotide evolution under the AIC using jModelTest v.2.1.4 software (Darriba et al., 2012). The model of substitution used was GTR+I+G for 397 bp mtDNA CR.

The second tree included all individuals that amplified 7-mtDNA regions (*n* = 40). We aligned all concatenated sequences obtained with 66 concatenated sequences from 7-mtDNA regions available in the GenBank (65 belonging to different species of *Tursiops* and one as outgroup). The 65 concatenated sequences from 7-mtDNA regions belong to *T. truncatus* (*n* = 59), *T. aduncus* (*n* = 4), Burrunan dolphin (“*T. australis*”, *n* = 2). The details of the mtDNA regions and their corresponding GenBank accession number are summarized in Table S5. One outgroup was the rough-toothed dolphin, *S. bredanensis* (accession number JF339982.1). We aligned all sequences using CLUSTAL W implemented in MEGA 6 software (Tamura et al., 2013). Given the mitogenome of *S. bredanensis* was incomplete, the analyses were based on 5,209 bp truncated alignment. The concatenated consensus sequence obtained was edited and checked visually. The concatenated sequences matrix is detailed in Supplemental Information 2. We analyzed concatenated sequences based on three partitioning schemes and applied the substitution model onto non-coding (the two partial ribosomal RNA genes, and eight partial tRNA genes), mtDNA CR, and protein-coding regions. We inferred the best-fitting model for nucleotide evolution under the AIC using jModelTest v.2.1.4 software (Darriba et al., 2012). The model of substitution used was TPM1uf+I, TPM3uf+I+G, and TVM+I+G for non-coding partitions, mtDNA CR, and protein coding regions, respectively.

Posterior probabilities of the trees and parameters in the evolutionary model were approximated with MCMC. Two independent runs of four chains were carried out to 10,000,000 and 20,000,000 generations with a 100,000 and 200,000 burn-in, sampling every 5,000 generations. We evaluated the effective sample size (ESS > 200) values using Tracer v.1.6 software (Rambaut et al., 2014) to ensure mixing and convergence of the posterior distribution and parameters. In addition, we examined the values of potential scale reduction factor (PSRF = 1) and the average standard deviation of split frequency between chains (≤ 0.01). We visualized and edited the phylogenetic tree using FigTree v.1.4.2 (Rambaut, 2015).

## Results

Of the 29 bone samples included in the study, 13 samples could not be amplified for mtDNA CR (Table S1). Sex was determined only for 36 individuals. All bone samples (*n* = 29) and four skin samples could not be amplified. The five stranded samples were identified as four females and one male. In estuarine bottlenose dolphins, molecular sex determination showed a sampling bias in favor of males (22) over females (9) (sex ratio 2.4:1). The details are summarized in Table S1.

### Genetic diversity

**Figure 2:**
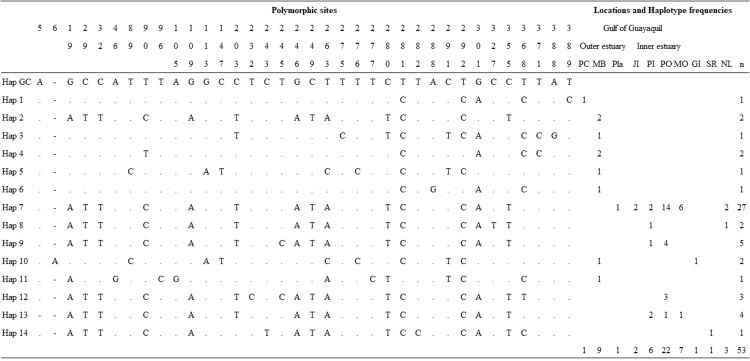
Mitochondrial control region haplotype polymorphic nucleotides and haplotype frequencies in the Gulf of Guayaquil. Polymorphic sites and haplotype frequency identified within a 392 bp sequence. The identity of the sequences was compared to the coastal haplotype from the Gulf of California (Hap GC, GenBank accession number KF570389.1). Numbers in the heading row indicate the base pair position of the polymorphic sites. Dots (.) show the identity with the Hap GC and the dash (-) represents a gap or deletion. PC, Punta Carnero-Salinas; MB, Mar Bravo-Salinas; Pla, Playas; JI, Jambelí Island; PI, east part of Puná Island; PO, Posorja; MO, El Morro; GI, Galápagos Islands; SR, Santa Rosa-Peru; NL, No location; n, number of samples by haplotype.

Overall, we obtained three mtDNA CR fragments of 694 (*n* = 40), 450 (*n* = 13), and 350 bp (*n* = 3), which were truncated to 392 bp. The sequences shorter than 392 bp were excluded from all analyses. A total of 48 samples were analyzed for the genetic diversity of Gulf of Guayaquil dolphin populations. Overall, haplotype diversity (*h*) and nucleotide diversity (π) were *h* = 0.7154 ± 0.0676 and π = 0.1510 ± 0.0826, respectively. mtDNA CR genetic diversity was higher in the outer estuary population than in the inner estuary population (Table 1). The analysis indicated 32 polymorphic sites, which revealed 13 distinct haplotypes not previously described for 392 bp mtDNA CR (Fig. 2; Table S6). Haplotypic diversity varied between two geographic areas and between three inner estuary locations. The number of haplotypes in the inner estuary population was lower than the outer estuary population. Eight haplotypes (Hap 1–Hap 6, Hap 10, and Hap 11) were detected in the outer estuary population: one in a sample from Punta Carnero and the others for samples from Mar Bravo. Five (Hap 7–Hap 9, Hap 12, and Hap 13) haplotypes were revealed for the inner estuary population. One haplotype was shared among the sites at Playas, Posorja, the east side of Puná Island, and the El Morro (Hap 7). Two haplotypes were shared among inner estuary communities: one between Posorja, the east side of Puná Island, and the El Morro (Hap 13), and the other with Posorja and the east side of Puná Island (Hap 9). Two were unique haplotypes, one at Posorja (Hap 12) and one on the east side of Puná Island (Hap 8) (Fig. 2).

**Table 1:** Genetic diversity indices based on 392 bp mtDNA CR sequence for each population of the Gulf of Guayaquil.

Locations	*n*	H	S	Hd	π	Tajima’s D	Fu’s Fs
Mar Bravo	9	7	29	0.9444 ± 0.0702	0.3400 ± 0.1936	0.3387 ns	0.5223 ns
Punta Carnero	1	1	–	–	–	–	–
Playas	1	1	–	–	–	–	–
**Overall outer estuary**	11	9	29	0.9636 ± 0.0510	0.0320 ± 0.1784	0.3860 ns	−0.6708 ns
El Morro	7	2	1	0.2857 ± 0.1964	0.0089 ± 0.0122	0.00 ns	−0.0947 ns
East side of Puná Island	6	4	3	0.8667 ± 0.1291	0.0375 ± 0.0318	−1.1319 ns	−1.4544 ns
Posorja	22	4	3	0.5671 ± 0.1038	0.0240 ± 0.0207	0.6304 ns	−0.4431 ns
Jambelí Island	2	1	–	–	–	–	–
**Overall inner estuary**	37	5	3	0.5571 ± 0.0864	0.0228 ± 0.0191	−0.5045 ns	−1.1606 ns
**Overall Gulf of Guayaquil**	48	13	32	0.7154 ± 0.0676	0.1510 ± 0.0826	−1.0611 ns	−0.0629 ns

**Notes:**

*n*, number of samples by population; S, polymorphic sites; H, number of haplotypes; Hd, haplotype diversity; π, nucleotide diversity; ns, no significant.

### Population structure

On a smaller spatial scale, the AMOVA performed with mtDNA CR showed a moderate genetic differentiation between both outer and inner estuary populations (mtDNA CR: percentage of variation among groups = 23.11%, *F*_ST_ = 0.2311, *P* < 0.05). However, the analysis of genetic distances (φ_ST_) did not indicate differences between locations (CR region: percentage of variation among groups = 0%, φ_ST_ = 0, *P* = 1). On a broad geographic scale, the analyses indicated a genetic structuring among populations (mtDNA CR: percentage of variation among groups = 6.20%, *F*_ST_ = 0.0619, *P* < 0.05) and genetic distances (percentage of variation among groups = 39.54 %, *F*_ST_ = 0.3954, *P* < 0.05). Pairwise *F*_ST_ and φ_ST_ comparisons confirmed significant differences among estuarine bottlenose dolphin population and other bottlenose dolphin populations; whereas, the outer estuary population was significantly different from the majority of the dolphin population (Table 2).

**Table 2:** Pairwise *F*_ST_ and φ_ST_ distance for global common bottlenose dolphin populations.

Strata	Ecotype	*n*	H	Pairwise distance				AMOVA		Reference
				Inner estuary		Outer estuary		*F*_ST_	φ_ST_	
				*F*_ST_	φ_ST_	*F*_ST_	φ_ST_			
**Pacific Ocean**										
GG-Inner estuary	C	37	5	–	–	0.2310*	0.6979*	0.0794	0.4219	This study
GG–Outer estuary	U	11	9	0.2310*	0.6979*	–	–	0.0627	0.3808	This study
Gulf of California	U	40	37	0.2211*	0.7058*	0.0188*	0.0639*	0.079	0.3883	1, 2, 3
Gulf of California	C	19	13	0.2095*	0.8748*	0.0387*	0.0796*	0.058	0.4071	1, 2, 3
Gulf of California	O	62	55	0.2634*	0.6621*	0.0186*	0.1160*	0.057	0.3848	1, 2, 3
Hawaiian Islands	U	26	26	0.2327*	0.7355*	0.0170*	0.1699*	0.061	0.3780	4
China	C	16	9	0.3341*	0.8932*	0.1134*	0.4425*	0.058	0.3989	5
Japan	U	22	21	0.2406*	0.8207*	0.0195*	0.3449*	0.068	0.3886	6, 7
**Atlantic Ocean**										
Eastern North Atlantic	C	9	7	0.2976*	0.8994*	0.0457*	0.2577*	0.064	0.4013	8
Eastern North Atlantic	P	11	10	0.2734*	0.8732*	0.0272	0.2295*	0.061	0.3980	8
Eastern North Atlantic	C	10	10	0.2693*	0.8781*	0.0183	0.2235*	0.061	0.3985	9
Scotland	C	8	3	0.4265*	0.9710*	0.2030*	0.4677*	0.041	0.4232	9, 10
Ireland	C	12	12	0.2617*	0.8508*	0.0180*	0.2177*	0.060	0.3930	11
Azores	O	82	37	0.2184*	0.7252*	0.0345*	0.3286*	0.058	0.3936	12
Portugal	O	13	10	0.2818*	0.8555*	0.0439*	0.2489*	0.062	0.3953	12
Madeira	O	25	15	0.2618*	0.8284*	0.0472*	0.3057*	0.061	0.3998	12
Western North Atlantic	C	9	8	0.2858*	0.9542*	0.0321	0.5842*	0.063	0.4132	10
Western North Atlantic	P	9	9	0.2739*	0.8824*	0.0186	0.2609*	0.062	0.3935	10
Bahamas	C	11	11	0.2653*	0.8608*	0.0181	0.3869*	0.061	0.3897	13, 14
**Mediterranean Sea**										
East Mediterranean	C	20	15	0.2571*	0.8279*	0.0338*	0.2771*	0.060	0.3959	9, 10
West Mediterranean	C	12	12	0.2617*	0.8730*	0.0180*	0.2545*	0.060	0.3968	9
**Black Sea**	C	16	12	0.2699*	0.8775*	0.0391*	0.3885*	0.061	0.3985	9, 10

**Notes:**

Number of samples by population (*n*), number of haplotypes (H). GG, Gulf of Guayaquil; C, Coastal; O, offshore; U, unknown. Statistically significant comparisons are marked by asterisks (*P* < 0.05 = *). References: (1) Segura et al. (2006) (DQ105702.1–DQ105733.1); (2) Segura-García et al. (2018) (HE617258.1–HE617294.1, HE617296.1, HE617297.1); (3) Perrin et al. (2011) (HQ206659.1–HQ206682.1, HQ206684.1–HQ206714.1); (4) K. Martien & K. Robertson (2008, unpublished data) (EF672700.1–EF672725.1); (5) G. Ji et al. (2001, 2002, unpublished data) (AF355582.1–AF355587.1, AF459508.1–AF459515, AF459522.1, AF459523.1); (6) S. Kitamura & S. Abe (2013, unpublished data) (AB610376.1); (7) Kita et al. (2013) (AB303154.1–AB303174.1); (8) M. Nykanen & A. Foote (2016, unpublished data) (KT601188.1–KT601207.1); (9) Natoli, Peddemors & Hoelzel (2004) (AY963588.1–AY963626.1); (10) Moura et al. (2013) (KF570316.1–KF570334.1, KF570345.1–KF570352.1, KF570370.1–KF570389.1); (11) Mirimin et al. (2011) (HQ634245.1–HQ634251.1, HQ634253.1–HQ634257.1); (12) Quérouil et al. (2007) (DQ525357.1–DQ525388.1, DQ073641.1–DQ073672.1, DQ073674.1–DQ073718.1, DQ073720.1–DQ073729.1, GQ241419.1); (13) K. Parsons et al. (2001, unpublished data) (AF378176.1–AF378178.1); (14) Parsons et al. (1999, 2006) (AF155160.1–AF155162.1; DQ118180.1–DQ118184.1).

### Population divergence analyses

The number of net nucleotide substitutions per site (*d_A_*) and the average divergence (*d_XY_*) values showed a divergence (*d_A_* = 0.015 and *d_XY_* = 0.030) between both outer and inner estuary populations. At the same time, the population divergence analyses revealed a high divergence between the estuarine bottlenose dolphin population and all other populations. The *d_A_* values varied between 0.023 and 0.047 and the *d_XY_* values varied between 0.034 and 0.052. Additionally, the analyses of population divergence did not indicate divergence between the outer estuary population and some bottlenose dolphin populations, particularly with Gulf of California populations. The *d_A_* values varied between 0.001 and 0.038 and *d_XY_* values varied between 0.022 and 0.044 (Table 3).

**Table 3:** Calculations of average evolutionary divergence between different geographic locations (*d*_XY_) and the number of net nucleotide substitution per site between populations (*d*_A_).

Strata	E	*n*	H	No. of bp	*d_A_*		*d_XY_*		Reference
					Inner	Outer	Inner	Outer	
**Pacific Ocean**									
GG-Inner estuary	C	37	5	397	–	0.015	–	0.030	This study
GG–Outer estuary	U	11	9	397	0.015	–	0.030	–	This study
Peru	O	9	9	317*	0.032	0.003	0.045	0.029	1
Gulf of California	U	40	37	397	0.028	0.001	0.040	0.027	2, 3, 4
Gulf of California	C	19	13	397	0.030	0.002	0.037	0.022	2, 3, 4
Gulf of California	O	62	55	397	0.029	0.002	0.042	0.028	2, 3, 4
Hawaiian Islands	U	26	26	397	0.033	0.005	0.048	0.034	5
China	C	16	9	397	0.043	0.015	0.052	0.038	6
Japan	U	22	21	397	0.038	0.038	0.050	0.037	7, 8
New Zealand	C	25	21	360	0.027	0.005	0.042	0.033	9, 10
Melanesia	U	18	15	360	0.032	0.011	0.044	0.035	11
Australia	C	5	5	360	0.031	0.008	0.043	0.033	12
**Atlantic Ocean**									
Eastern North Atlantic	C	9	7	397	0.033	0.008	0.041	0.030	13
Eastern North Atlantic	P	11	10	397	0.031	0.007	0.041	0.030	13
Eastern North Atlantic	C	10	10	397	0.030	0.007	0.040	0.030	14
Scotland	C	8	3	397	0.040	0.016	0.041	0.031	14, 15
Ireland	C	12	12	397	0.031	0.007	0.042	0.041	16
Azores	O	82	37	397	0.032	0.008	0.042	0.031	17
Portugal	O	13	10	397	0.032	0.007	0.042	0.031	17
Madeira	O	25	15	397	0.033	0.009	0.043	0.032	17
United Kingdom	U	13	11	360	0.026	0.026	0.035	0.030	18
Western North Atlantic	C	9	8	397	0.047	0.025	0.051	0.044	15
Western North Atlantic	O	9	9	397	0.033	0.008	0.045	0.033	15
Bahamas	C	11	11	397	0.034	0.015	0.046	0.041	19, 20
Florida	C	7	7	360	0.024	0.010	0.036	0.034	21
Caribbean Sea	C	11	11	360	0.029	0.020	0.036	0.040	22, 23
Caribbean Sea	W	12	12	360	0.023	0.007	0.036	0.033	22
Mid-Atlantic	O	17	2	360	0.033	0.014	0.034	0.029	24
**Mediterranean Sea**									
East Mediterranean	C	20	15	397	0.032	0.008	0.042	0.031	14, 15
West Mediterranean	C	12	12	397	0.033	0.007	0.043	0.031	14
**Black Sea**	C	16	12	397	0.038	0.013	0.048	0.035	14, 15

**Notes:**

Number of samples by population (*n*), number of haplotypes (H), number of base pairs (No. of bp), Ecotype (E). 317 bp mtDNA CR analysis included two stranded samples from Puná Island (*). GG, Gulf of Guayaquil; C, Coastal; O, offshore; U, unknown; W, worldwide distribution. References: (1) A. Barreto et al. (2006, unpublished data) (AF323893.1, AF323895.1, AF323897.1–AF323903.1); (2) Segura et al. (2006) (DQ105702.1–DQ105733.1); (3) Segura-García et al. (2018) (HE617258.1–HE617294.1, HE617296.1, HE617297.1); (4) Perrin et al. (2011) (HQ206659.1–HQ206682.1, HQ206674.1–HQ206714.1); (5) K. Martien & K. Robertson (2008, unpublished data) (EF672700.1–EF672725.1); (6) G. Ji et al. (2001, 2002, unpublished data) (AF355582.1–AF355587.1, AF459508.1–AF459515, AF459522.1, AF459523.1); (7) S. Kitamura & S. Abe (2013, unpublished data) (AB610376.1); (8) Kita et al. (2013) (AB303154.1–AB303174.1); (9) Tezanos-Pinto et al. (2009) (EU276389.1–EU276412); (10) Caballero et al. (2008) (EU1221118.1); (11) Oremus et al. (2015) (KF555574.1–KF555591.1); (12) Charlton-Robb et al. (2011) (JN571470.1–JN571474.1); (13) M. Nykanen & A. Foote (2016, unpublished data) (KT601188.1–KT601207.1); (14) Natoli, Peddemors & Hoelzel (2004) (AY963588.1–AY963626.1); (15) Moura et al. (2013) (KF570316.1–KF570334.1, KF570345.1–KF570352.1, KF570370.1–KF570389.1); (16) Mirimin et al. (2011) (HQ634245.1–HQ634251.1, HQ634253.1–HQ634257.1); (17) Quérouil et al. (2007) (DQ525357.1–DQ525388.1, DQ073641.1–DQ073672.1, DQ073674.1–DQ073718.1, DQ073720.1–DQ073729.1, GQ241419.1); (18) V. Islas-Villanueva et al. (2017, unpublished data) (KP967565.1–KP967576.1); (19) K. Parsons et al. (2001, unpublished data) (AF378176.1–AF378178.1); (14) Parsons et al. (1999, 2006) (AF155160.1–AF155162.1; DQ118180.1–DQ118184.1). (21) Lewis et al. (2013) (KC121569.1–KC121575.1); (22) Caballero et al. (2012) (JN596281.1–JN596289.1, JN596293.1, JN596297.1–JN596304.1, JN596312.1, JN596216.1, JN596318.1, JN596319.1); (23) Barragán-Barrera et al. (2017) (KX833116.1); (24) Castilho et al. (2015) (KC896604.1–KC896620.1).

### mtDNA CR phylogeographic and phylogenetic analyses

**Figure 3:**
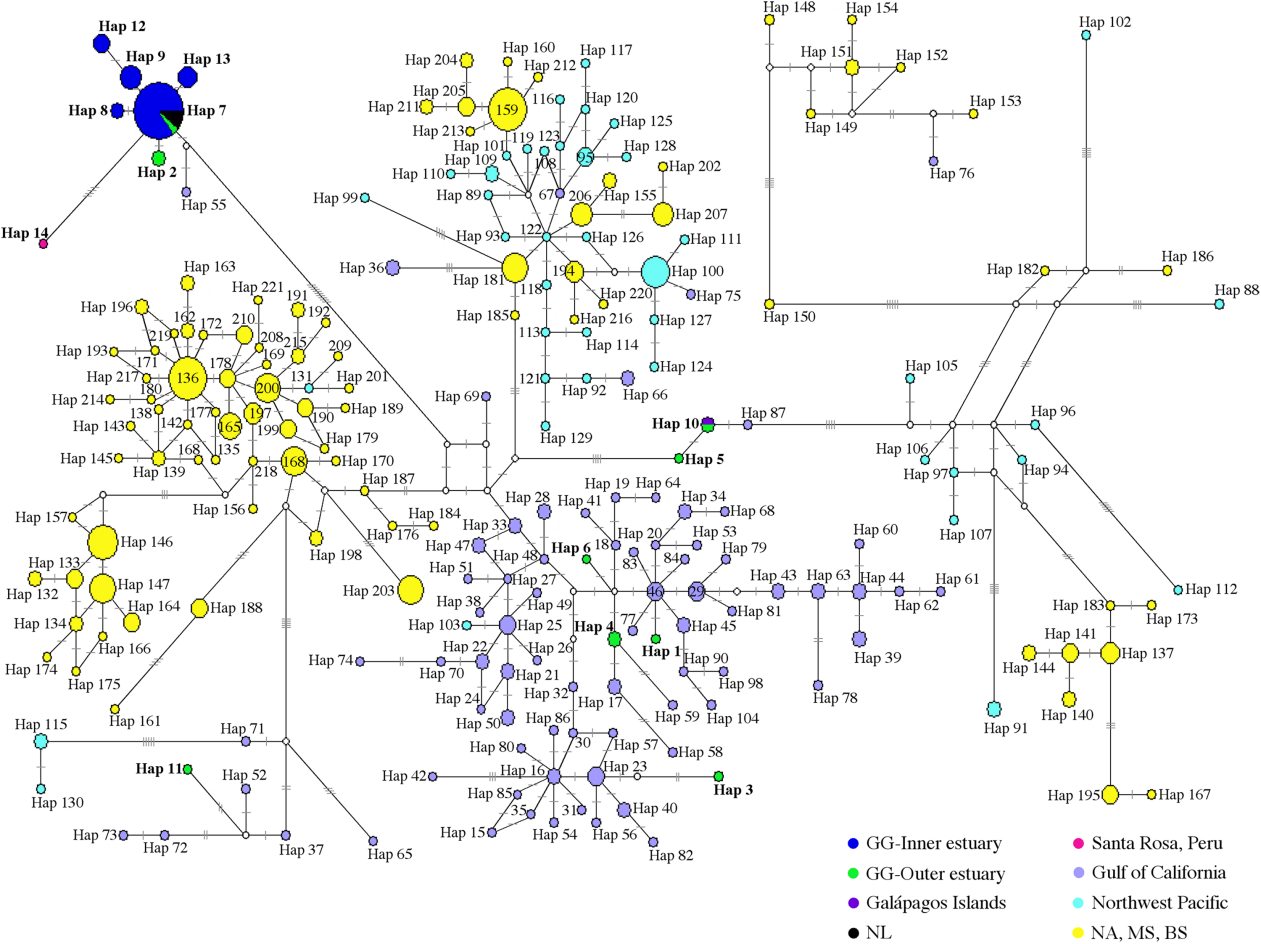
Median-joining network of common bottlenose dolphin mtDNA CR. Circle size is related to the number of individuals that share the same haplotype. The circles are colored according to the geographic region shown in the legend. The white circle corresponds to the missing or intermediate haplotype. The length of the branch is proportional to the number of mutational steps among haplotypes. Hatch marks show the total number of mutations between haplotypes. GG, Gulf of Guayaquil; NL, No location; NA, North Atlantic; MS, Mediterranean Sea; BS, Black Sea.

**Figure 4:**
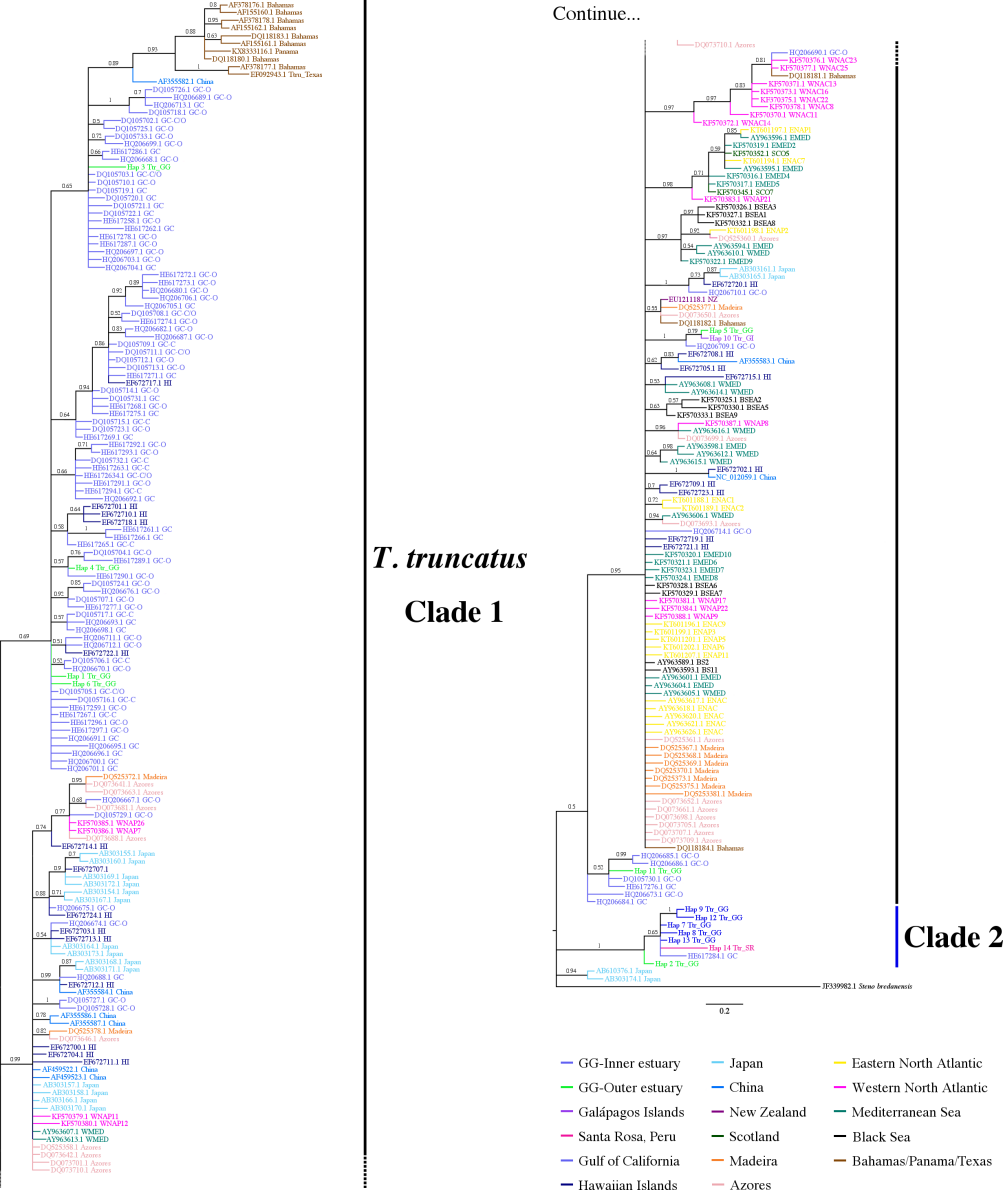
Bayesian phylogenetic tree showing the genetic divergence between sequences of the mtDNA CR of the estuarine bottlenose dolphin and other analyzed regions. Bayesian phylogenetic tree inferred from the analysis of 397 bp mtDNA CR sequences. Numbers above the main branches represent posterior probability values. The names of the sequences are colored according to the geographic region shown in the legend. The names of the sequences obtained from GenBank are labeled with their location and accession number. Clade 1 is conforming principally by sequences from different geographic areas obtained from GenBank. Clade 2 is composed exclusively of haplotipes obtained in this study. Outgroup includes the harbor porpoise (*Steno bredanensis*). Hap, haplotype; Ttr, *Tursiops truncatus*; GG, Gulf of Guayaquil; GI, Galápagos Islands; SR, Santa Rosa-Peru; GC, Gulf of California; HI, Hawaiian Islands; NZ, New Zealand; ENAC, Eastern North Atlantic Coastal; ENAP, Eastern North Atlantic Pelagic; WNAC, Western North Atlantic Coastal; WNAP, Western North Atlantic Pelagic; BSEA/BS, Black Sea; WEMED, Western Mediterranean; EMED, Eastern Mediterranean; SCO, Scotland.

Of 53 of 397 bp mtDNA CR sequences, we identified 14 haplotypes (Fig. 2; Table S6). The median-neighbor joining method showed the relationships among haplotypes from different populations of bottlenose dolphins, revealing evidence of a genetic divergence between estuarine bottlenose dolphin and North Pacific/Atlantic Ocean dolphin populations. The network analysis indicated that the group of haplotypes from the free-ranging dolphins plus the haplotypes from the stranded dolphins collected at different sites in the Gulf of Guayaquil (Hap 2, Hap 8, Hap 9, Hap 12, and Hap 13) and one from Peru (Hap 14) clustered together and diverged from the other haplotypes of different geographic locations. The main characteristic of this group was the presence of a central haplotype (Hap 7) mainly consisting of samples from the Gulf of Guayaquil inner estuary. The central haplotype was connected to other haplotypes from the inner estuary, and one from Peru. The other Gulf of Guayaquil haplotypes (Hap 1, Hap 3–6, and Hap 11) were closely related to other dolphin populations from the northeast Pacific. The Hap 10, comprising one sequence from the Galápagos Islands and another from Mar Bravo, was closely related to the offshore ecotype haplotype. Additionally, the network revealed several mutational steps (∼10) away from the main group, indicating a divergence of haplotypes (Fig. 3). Overall, the median-joining network method was consistent, demonstrating a close relationship among haplotypes of the 397 bp mtDNA CR of estuarine bottlenose dolphins, signifying phylogeographic separation. The pattern of genetic divergence is in agreement with the Bayesian inference of 397 bp mtDNA CR. A phylogenetic Bayesian inference tree built using mtDNA CR sequences showed a genetic divergence between the estuarine bottlenose dolphin and the other regions analyzed, seeing as all estuarine bottlenose dolphin haplotypes formed a single clade with a probability value of 1 (Fig. 4).

### 7-mtDNA regions phylogenetic analyses

Overall, we obtained a 5,237 bp concatenated consensus sequence of 7-mtDNA regions: (i) 12S rRNA-16S rRNA of 1,050 bp (*n* = 40), (ii) 16S rRNA of 562 bp (*n* = 47), (iii) ND1-ND2 of 897 bp (*n*= 40), (iv) COI of 837 bp (*n* = 40), (v) COII of 738 bp (*n* =40), (vi) *Cyt b* of 436 bp (*n* = 47), and (vii) CR of 715 bp (*n* = 45). In Table S7 is detailed haplotypes per region, per sample, and the accession number of the sequence that has a high percent of similarity. The samples that did not amplify for all 7-mtDNA regions were excluded from the phylogenetic analyses. The analyses were based on 5,209 bp truncated alignment. Of 40 concatenated sequences, we identified 28 haplotypes: (i) 26 unique mtDNA concatenated haplotypes in the Gulf of Guayaquil (20 from the Gulf of Guayaquil inner estuary, and six from the Gulf of Guayaquil outer estuary), (ii) one from the Galápagos Islands, and (iii) one from Santa Rosa, Peru.

**Figure 5:**
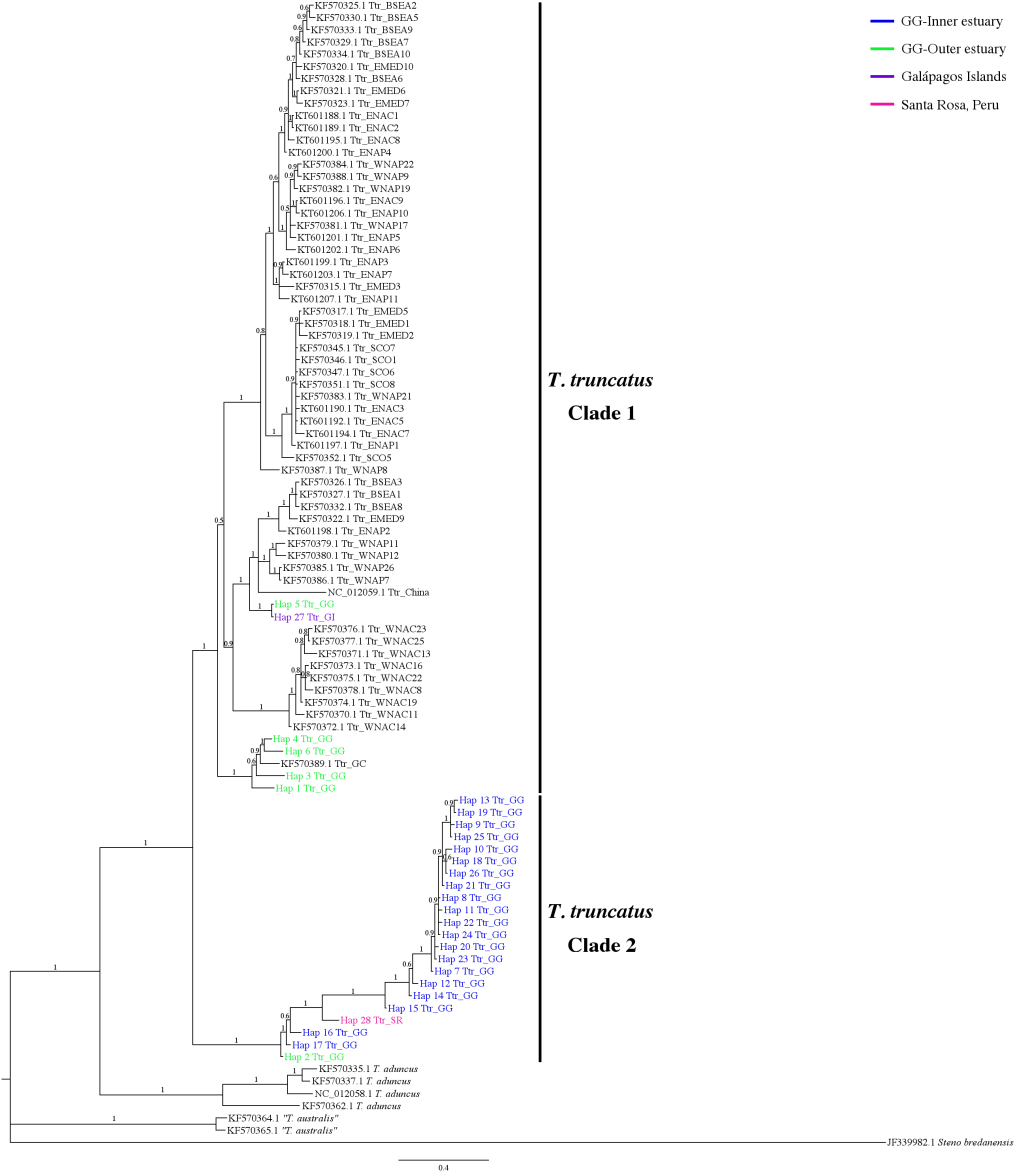
Bayesian phylogenetic tree showing the genetic divergence between the estuarine bottlenose dolphin and other populations elsewhere. Bayesian phylogenetic tree inferred from the analysis of a 5,209 bp concatenated sequence of 7-mtDNA regions. Numbers above the main branches represent posterior probability values. The names of the sequences obtained in the present study are colored according to the geographic region shown in the legend. The names of the sequences obtained from GenBank are labeled with their location and accession number. Clade 1 is conforming principally by sequences from different geographic areas obtained from GenBank. Clade 2 is composed exclusively of haplotypes obtained in this study. Outgroup includes rough-toothed dolphin (*Steno bredanensis*). Hap, haplotype; Ttr, *Tursiops truncatus*; GG, Gulf of Guayaquil; GI, Galápagos Islands; SR, Santa Rosa-Peru; GC, Gulf of California; WNAC, Western North Atlantic Coastal; WNAP, Western North Atlantic Pelagic; BSEA, Black Sea; EMED, Eastern Mediterranean; SCO, Scotland; ENAC, Eastern North Atlantic Coastal; ENAP, Eastern North Atlantic Pelagic.

The 7-mtDNA regions from the 94 haplotypes presented 550 variable sites, of which 345 were parsimony informative sites and 18 included gaps. The details of sequence variation and tree characteristics are shown in Table S8. The Bayesian inference produced a well-defined and strongly supported tree with posterior probability values > 0.9 for most nodes (Fig. 5). All *T. truncatus* sequences formed a single clade with a probability value of 1. The *T. truncatus* is divided into two well-supported clades 1 and 2. Clade 1 is composed principally of coastal and offshore ecotypes from different geographic areas, including five haplotypes from the stranded dolphins. Four haplotypes from stranded dolphins (Hap 2: Ttr_2 from Punta Carnero, Hap 3: Ttr_4, Hap 4: Ttr_5, and Hap 6: Ttr_7 from a location in Mar Bravo) were clustered with the single coastal haplotype from the Gulf of California (accession number KF570389.1), whereas two haplotypes (from the Galápagos Islands, Hap 27: Ttr_1, and the Mar Bravo location, Hap 5: Ttr_6) were clustered with the sequences of the pelagic population from Western Atlantic Ocean as well as with sequences of the coastal populations from the Black Sea, and the Mediterranean Sea (Fig. 5). Clade 2 is composed exclusively of haplotypes obtained in the present study: 20 haplotypes from the free-ranging dolphins of the inner estuary (Hap 7–Hap 26) and two haplotypes from stranded animals (from Peru, Hap 28: Ttr_69, and Hap 2: Ttr_3 and Ttr_8 from a location in Mar Bravo), grouping them as a monophyletic group. Overall, the phylogenetic analyses showed that the dolphin population of the Gulf of Guayaquil inner estuary has a different evolutionary pattern compared with different *T. truncatus* populations.

## Discussion

To our knowledge, this is the first study that has assessed the evolutionary link between the estuarine bottlenose dolphin in the Gulf of Guayaquil and *T. truncatus* populations elsewhere using analysis of several mtDNA regions. Our results showed a genetic differentiation and divergence between estuarine bottlenose dolphins and other *T. truncatus* populations from other parts of the species’ range. mtDNA CR diversity was lower in estuarine bottlenose dolphins than in the outer bottlenose dolphin population. No shared mtDNA CR haplotypes were found between estuarine bottlenose dolphins and other dolphin populations, suggesting a long-term historical separation. These results were consistent with phylogenetic analysis from different mtDNA regions, revealing that the estuarine bottlenose dolphin population has had a distinct evolutionary history. The findings from the inclusion of a single sample from Peru show it is closely related to the estuarine bottlenose dolphin, implying that both south east Pacific coastal populations are divergent from the entire analyzed *T. truncatus* population. However, these results should be accepted with caution because we used a small sample size and only one molecular marker. It is important to include both nuclear and mitochondrial markers to assure reliable results in addition to a sufficient number of samples that truly represent the population size of the study area. We acknowledge the small sample sizes and the matrilineal marker used for the current study may not have been sufficient to conduct reliable genetic analyses for the Gulf of Guayaquil bottlenose dolphins. However, it is important to note the coastal bottlenose dolphins generally occur in small populations and an estimate of the actual dolphin population size in the Gulf of Guayaquil is not available. Nevertheless, further work should be done to increase sample sizes as well as the number of molecular markers to corroborate our conclusions.

### Low genetic diversity of the estuarine bottlenose dolphin

The low level of mtDNA CR genetic diversity of both outer and inner estuary dolphin populations are similar to those observed in coastal dolphins (Natoli, Peddemors & Hoelzel, 2004; Quérouil et al., 2007; Tezanos-Pinto et al., 2009; Caballero et al., 2012) and estuarine resident populations (Rosel, Hansen & Hohn, 2009). These results are in agreement with those described from 29 samples from northeastern Scotland and other regions from the UK and Ireland, where the genetic diversity showed similar values (*h* = 0.697) (Parsons et al., 2002). The genetic diversity reported for the outer population is comparable to those documented in offshore populations from the northeast Atlantic, Brazil, and the Gulf of California (Quérouil et al., 2007; Louis et al., 2014a; Fruet et al., 2014; Lowther-Thieleking et al., 2015). The low genetic diversity detected in the estuarine bottlenose dolphin population could be due to the small number of resident dolphins (45 and 70) that inhabit a small, restricted area (Jiménez & Alava, 2014; Félix et al., 2017a). Similar levels of genetic diversity were previously detected in “inshore” Caribbean populations (*h* = 0.578, π = 0.9) (Caballero et al., 2012). The low level of genetic diversity detected in the estuarine resident populations may result from a single or compounded situations: (i) small population size (Richards et al., 2013); (ii) a founder event (Oremus et al., 2015); (iii) younger populations moving from offshore to coastal locations (Parsons et al., 2002); or (iv) a complete absence of maternal gene flow from neighboring populations (Richards et al., 2013). Low nucleotide diversity values found in the present study are similar to those found in populations that have experienced a population bottleneck (Luikart & Cornuet, 1998). For instance, a strong reduction of genetic diversity has been previously reported in other cetaceans, such as *Orcinus orca* (Moura et al., 2014) and *T. aduncus* (Oremus et al., 2015; Amaral et al., 2017). However, Tajima’s and Fu’s Fs selective neutrality tests do not support the population bottleneck hypothesis, but these tests can be affected by a sample size (*n* = 31) (see Subramanian, 2016).

### Population structure in Gulf of Guayaquil dolphins

A moderate but significant genetic structure was observed in the outer and inner estuary populations (*F*_ST_ = 0.231, *P* < 0.05). The pattern of genetic differentiation among bottlenose dolphins inhabiting adjacent habitat types has been seen previously in populations of *T. truncatus* (Tezanos-Pinto et al., 2009; Fruet et al., 2014, 2017). In particular, *T. truncatus* coastal dolphin populations present genetic differentiation at fine geographic scale in different parts of the world (Rosel, Hansen & Hohn, 2009; Tezanos-Pinto et al., 2009; Mirimin et al., 2011; Caballero et al., 2012). Similarly, the same levels of population structure have also been reported for two communities of coastal *T. truncatus* in Ireland (Mirimin et al., 2011). The population differentiation may be explained by the estuarine dolphins local site fidelity (Jiménez & Alava, 2014; Félix et al., 2017a), as well as the geographic differences between the outer and inner estuaries (see Stevenson, 1981; Twilley et al., 2001). These results suggest that a historical genetic separation between both populations together with ecological factors may promote divergence, resulting in fine-scale population structure (Wiszniewski et al., 2014). Although common bottlenose dolphins are capable of traveling long distances, the estuarine bottlenose dolphin may remain confined to small geographic areas.

On a broad scale, our results indicated that the estuarine bottlenose dolphins are genetically differentiated from common bottlenose dolphin populations elsewhere. Indeed, the *d_A_* values above 0.02 showed a high genetic divergence, which could be interpreted as a subspecies. The phylogenetic and phylogeographic analyses support the genetic divergence that could be explained by a possible absence of historical interactions between populations. The presence of unique haplotypes and the mutational steps of the estuarine bottlenose dolphin suggests a long-term historical separation of the populations. Differentiation of estuarine bottlenose dolphins could have begun due to ocean re-structuring and climate variations (Steeman et al., 2009) after the opening of the Gulf of Guayaquil and the formation of the Morro Channel in the last interglacial period (Gutscher et al., 1999). The new geography of the inner estuary could provide a refuge to protect the dolphins from predators as well as serve as a nursery area. The special characteristics of the estuary, which include a level of ecological diversity with a network of islands and channels, sand and mud banks, and sandy beaches, as well as a high tidal range of 2–3 m, among other features, could have driven genetic differentiation and adaptive divergences, as has occurred with bottlenose dolphins (see Natoli, Peddemors & Hoelzel, 2004; Rosel, Hansen & Hohn, 2009; Fruet et al., 2014). In this new environment, the estuarine bottlenose dolphin was capable of specializing in different specific prey types and adopting specific foraging strategies limited to its home range in the Gulf of Guayaquil inner estuary (Jiménez & Alava, 2014; Félix et al., 2017a). Finally, a rapid divergence of the estuarine population may be the result of the low carrying capacity of estuaries as proposed for coastal populations (Gaspari et al., 2015).

It has been suggested that limited gene exchange supports genetic variation and isolation that may lead to speciation and endemism (Natoli, Peddemors & Hoelzel, 2004; Natoli et al., 2005; Möller et al., 2007; Amaral et al., 2017). The possible lack of historical gene exchange between the estuarine bottlenose dolphin and other populations of *T. truncatus* could have occurred after a possible founder effect of coastal dolphins in embayment areas (Sellas, Wells & Rosel, 2005; Möller et al., 2007; Tezanos-Pinto et al., 2009). A similar pattern was proposed for the Mediterranean Sea subpopulations of *T. truncatus* (Moura et al., 2013). Founder events may play an important role in adaptation to new environments (Wiszniewski et al., 2014) with local specialized resources (Hoelzel, Potter & Best, 1998). It has been suggested that habitat specialization in *T. truncatus* occurred independently in different areas over a wide range of its distribution (Caballero et al., 2012) and was driven by environmental factors (Natoli et al., 2005), which explains diversification (Moura et al., 2013). Prey availability could be one of these environmental factors. The mangrove forests in the Gulf of Guayaquil and the oceanographic characteristics of the estuary (temperature and salinity) contribute to the high diversity of prey species (Stevenson, 1981; Twilley et al., 2001). This prey availability and distribution constitute a driving factor for shaping the pattern of dolphin distribution (Degrati et al., 2013). *d_A_* values above 0.02 permit distinguishing between subspecies (Rosel et al., 2017); however, additional molecular markers need to be analyzed before a formal distinction at the subspecies level can be considered (Taylor et al., 2017).

### Genetic divergence of the Gulf of Guayaquil estuarine bottlenose dolphin population

The 7-mtDNA regions data show a clear phylogenetic differentiation between estuarine bottlenose dolphins and *T. truncatus* populations from other parts of the species’ range. It is clear that the estuarine bottlenose dolphin followed an independent evolutionary trajectory, suggesting that this population may be a distinct lineage. The systematics of *T. truncatus* is not well-defined at the intraspecific level since bottlenose dolphins are part of a wider taxonomic problem that involves the entire Delphininae subfamily, particularly the STD complex (i.e., *Stenella*, *Tursiops*, and *Delphinus*). This is because of the high intraspecific diversity and low interspecific divergence observed in this subfamily (Reeves et al., 2004). The taxonomic problem of this subfamily is complicated further by the effects of hybridism and introgression (Amaral et al., 2012). For example, studies have suggested that coastal and offshore ecotypes could constitute different lineages of *T. truncatus* (Natoli, Peddemors & Hoelzel, 2004; Caballero et al., 2012; Moura et al., 2013). According to our results, the estuarine bottlenose dolphin showed a different evolutionary pattern compared to populations elsewhere, including four stranded samples from the Gulf of Guayaquil outer estuary, which were more related to dolphins from the Gulf of California. Furthermore, the estuarine bottlenose dolphins differ genetically from the offshore ecotype, because the Galápagos Islands haplotype was most closely related to North Atlantic offshore dolphin populations (KF570386.1). The Galápagos Islands dolphin populations were formerly described as an offshore ecotype based on morphological data (Palacios, Salazar & Day, 2004). The estuarine bottlenose dolphin population could be evolutionarily linked to the coastal bottlenose dolphins of the western coast of South America, in particular to Peruvian populations, since the single Peruvian haplotype was included in this clade. Differences between Peruvian and Ecuadorian coastal populations have been reported based on morphological and prey composition data (Van Waerebeek et al., 1990; Félix, 1994; Santillán, Félix & Haase, 2008; Félix et al., 2017c), suggesting different populations. Indeed, it has been suggested that the coastal Peruvian population is different from the coastal Chilean populations, the latter being more related to the offshore Peruvian and Chilean population (Sanino et al., 2005). This evolutionary pattern could be attributed to incomplete lineage sorting or hybridization events as proposed previously to explain the divergence between the species of the Family Delphinidae (Amaral et al., 2012). The relationships within this genus can be linked to the rapid radiation of the species of the Family Delphinidae (Wang, Riehl & Dungan, 2014) during the Pleistocene epoch (Moura et al., 2013). Particularly, the estuarine bottlenose dolphin population could have been confined to small areas within the inner estuary during glacial stages, as suggested by Louis et al. (2014b) for coastal populations in the northeastern Atlantic. During the interglacial stages, this new coastal population became adapted to the new habitat with specialized foraging and territorial behavior (Ebenhard, 1991; Hanski & Gilpin, 1991; Louis et al., 2014b), causing a possible historical isolation of the estuarine bottlenose dolphin from other populations of *T. truncatus*. This population may constitute a distinct population structured in semi-closed resident communities (Hanski & Gilpin, 1991) with an evident genetic divergence as a consequence of an early expansion.

### Conservation implications

This study provides baseline evidence of the presence of at least two populations with high mtDNA divergence that should be taken into account to improve the management and conservation of the dolphin population in the area. Common bottlenose dolphin populations that inhabit coastal environments are exposed to distinct anthropogenic activities that negatively affect the viability of dolphin populations (Arcangeli & Crosti, 2009; Reif et al., 2009; Daura-Jorge & Simões-Lopes, 2011). In particular, the population’s dependence on the estuarine habitat makes it very susceptible to local anthropogenic disturbances, affecting its ecological and evolutionary processes. In the Gulf of Guayaquil inner estuary, intense fishing, vessel traffic, tourism, dredging, water pollution, and habitat destruction have been identified as risks for the resident dolphin population (Félix, 1997; Van Waerebeek et al., 2007; Castro et al., 2012; Jiménez & Alava, 2014; Van Bressem et al., 2015; Félix et al., 2017a, 2017b) that may have both short- and long-term negative effects. Therefore, how the impact of human activities differs across species’ ranges should be considered in population management actions.

One main focus of conservation is the maintenance of divergent populations (Allendorf, Luikart & Aitke, 2013). The estuarine bottlenose dolphin population inhabits a unique environment that promotes the formation of different ecological niches, leading to the population’s local adaptation. Its adjustment to conditions in the Gulf of Guayaquil’s ecosystem has generated not only a genetic divergence but also a distinction in morphological (Santillán, Félix & Haase, 2008; Félix et al., 2017c) and prey composition (Félix, 1994; Van Waerebeek et al., 1990), suggesting that the estuarine population should be managed as a distinct reproductive unit. In addition to the population size showing patterns of reduction (Jiménez & Alava, 2014; Félix et al., 2017a), other aspects of major concern are low genetic diversity, its restricted geographic home, and its site fidelity, which can lead to an increased risk of threat or even extinction. These factors make this population more vulnerable to environmental, demographic, and genetic stochasticity. Therefore, management and conservation strategies need to be developed to protect this population before current activities cause irreversible damage.

The conservation status for the *T. truncatus* population from Gulf of Guayaquil should be re-evaluated taking into consideration its particular genetic distinctiveness. It is justifiable to manage the estuarine bottlenose dolphin as a discrete lineage in order to prioritize conservation efforts, and it should be managed separately from other populations of *T. truncatus* elsewhere. Our findings associated with the ecological adaptation in this unique environment support the hypothesis that this population should be considered a MU in order to guide short-term management strategies and safeguard its genetic diversity. Therefore, it is important to take into consideration the population’s geographic distribution and the variety of threats that affect each group on different levels throughout their distribution range. Although the sample size is small, our findings provide a molecular baseline for management as a separate unit; actions should not be delayed given that the estuarine bottlenose dolphin inhabits a small area and is vulnerable to increasing coastal stressors.

## Conclusion

The genetic differentiation between the estuarine bottlenose dolphin and other *T. truncatus* populations deserves further attention. The well-defined and strongly-supported cluster in the phylogenetic tree indicates the Gulf of Guayaquil estuarine bottlenose dolphin followed an independent evolutionary trajectory, a conclusion we made based on mtDNA and which carries with it important implications for conservation management. According to the *d_A_* values above 0.02, the estuarine bottlenose dolphin may be a subspecies; however, additional nuclear and morphological markers need to be analyzed to considerer the Gulf of the Guayaquil inner estuary population as different subspecies. In particular, further investigations are needed to clarify the relationships of different coastal and offshore bottlenose dolphin populations from the southeast Pacific Ocean, including a combination of environmental, genetic, ecological, and morphometric data. The estuarine dolphin population is facing specific anthropogenic threats; therefore, it is justifiable to manage this population as a separate MU. Faced with an uncertain future, the estuarine bottlenose dolphin population requires that strategies be developed to minimize the impact of human activities if its conservation is to be made a priority. Developing special management strategies is crucial at this time because the dolphin population presents a reduced abundance and has shown a decline in the past years (Jiménez & Alava, 2014; Félix et al., 2017a). It is important that conservation strategies include: monitoring the number of dolphins in the area, determining their current geographic distribution and anthropogenic threats, and establishing their relationships to other Ecuadorian coastal populations. In addition, improving conservation of the mangrove ecosystem, which constitutes a refuge not only for the dolphins but also for other species, is key for developing any future strategy.

## Supplemental Information

10.7717/peerj.4589/supp-1 Supplemental Information 1 Phylogeographic relationships analysis database. The database includes mtDNA CR sequences (397 bp) from different geographic regions made available in the GenBank database with 53 CR region sequences of this study.

10.7717/peerj.4589/supp-2 Supplemental Information 2 Phylogenetic analysis database. The database includes 28 Ecuadorian haplotypes and 66 concatenated sequences from 7-mtDNA regions available in the GenBank.

10.7717/peerj.4589/supp-3 Supplemental Information 3 Sample information. All bone powder was collected on January 19, 2013. Acronyms: Ttr: *Tursiops truncatus,* NA: not available, CR: control region, *COI*: cytochrome oxidase I, *COII:* cytochrome oxidase II, *Cyt b*: cytochrome b. F: female, M: male, a) genetic diversity b) population structure and genetic divergence, c) phylogeographic analysis, d) mtDNA CR phylogenetic analysis, e) 7-mtDNA regions phylogenetic analysis. * skin biopsy from free-ranging dolphin, ^+^ genes/regions that were excluded from the analyses, - did not amplify.

10.7717/peerj.4589/supp-4 Supplemental Information 4 Primers used in the present study for the amplification of seven mitochondrial DNA regions. Acronyms: bp: base pair, Ta: Temperature annealing, mM: milimolar, CR: control region, *COI*: cytochrome oxidase I, *COII*: cytochrome oxidase II, *Cyt b*: cytochrome b, *ND1-ND2*: NADH dehydrogenase subunit I-II.

10.7717/peerj.4589/supp-5 Supplemental Information 5 List of mtDNA CR sequences of *Tursiops* *truncatus* from different locations obtained from GenBank used for phylogeographic analysis. The table includes the accession numbers and sequences geographic location. Number of samples by haplotype (n). Acronyms: Hap: Haplotype, C: coastal, O: Offshore, P: pelagic, U: unknown.

10.7717/peerj.4589/supp-6 Supplemental Information 6 List of mtDNA CR sequences of *Tursiops* *truncatus* from different locations obtained from GenBank used for the phylogenetic analysis. The table includes the accession numbers, haplotype name, sequences geographic location, and ecotypes. Number of samples by haplotype (n). Acronyms: E: Ecotype, C: coastal, P: pelagic, O: offshore, U: unknown.

10.7717/peerj.4589/supp-7 Supplemental Information 7 List of mtDNA mitogenomes of different species obtained from GenBank used for the phylogenetic analysis. The table includes the accession numbers, sequences geographic location, ecotypes, and the species. Acronyms: C: coastal, P: pelagic, U: unknown.

10.7717/peerj.4589/supp-8 Supplemental Information 8 List of 392 bp mtDNA CR haplotypes identified in the present study. Number of samples by haplotype (n). Acronyms: Hap: Haplotype, Ttr: *Tursiops truncatus*, * samples excluded from genetic diversity, population structure, and genetic divergence analyses.

10.7717/peerj.4589/supp-9 Supplemental Information 9 List of 7-mtDNA regions haplotypes identified in the present study. The table includes the accession numbers of the sequences which are similar to the sequences found in the present study. 100% of similarity is in bold. 95% of similarity is in red. Acronyms: H#: number of haplotype; Hap: Haplotype, Ttr: *Tursiops truncatus*, CR: Control region, *COI*: cytochrome oxidase I, *COII*: cytochrome oxidase II, *ND1-ND2*: NADH dehydrogenase subunit I-II, *Cyt b*: cytochrome b.

10.7717/peerj.4589/supp-10 Supplemental Information 10 Sequence variation and tree characteristics of the combined phylogeny. Total number of sequences (n), base pair (bp), polymorphic sites (S), non-coding (*12S rRNA*, *16S rRNA*, and *tRNAs*), Protein-coding genes (*ND1-ND2*, *COI*, *COII*, and *Cyt b*).

## Additional Information and Declarations

### Field Study Permissions

The following information was supplied relating to field study approvals (i.e., approving body and any reference numbers): Field collections were approved by the Ministry of Environment of Ecuador (permit number 004-IC-FAU-DPG/MAE).

### DNA Deposition

The following information was supplied regarding the deposition of DNA sequences: The sequences were deposited in GenBank (accession numbers: KU991990–KU992299). https://www.ncbi.nlm.nih.gov/nuccore/?term=Bayas-Rea.

### Data Availability

The following information was supplied regarding data availability: The raw data are provided in Supplemental Dataset Files.
