# Supplementary material for: Genetic divergence and fine scale population structure of the common bottlenose dolphin (Tursiops truncatus, Montagu) found in the Gulf of Guayaquil, Ecuador
Source: PeerJ. 2018 Apr 9;6:e4589. doi: 10.7717/peerj.4589 (PMC5916226; doi:10.7717/peerj.4589)
Supplement: Supplemental Information 3 — All bone powder was collected on January 19, 2013. Acronyms: Ttr: Tursiops truncatus, NA: not available, CR: control region, COI: cytochrome oxidase I, COII: cytochrome oxidase II, Cyt b: cytochrome b. F: female, M: male, a) genetic diversity b) population structure and genetic divergence, c) phylogeographic analysis, d) mtDNA CR phylogenetic analysis, e) 7-mtDNA regions phylogenetic analysis. * skin biopsy from free-ranging dolphin, + genes/regions that were excluded from the analyses, - did not amplify. [file peerj-06-4589-s003.docx]

| **Sample ID** | | **Sampling location** | **Tissue type** | **Collection date** | **Molecular sex identification** | **Genes/regions amplified** | **Specific test** |
| --- | --- | --- | --- | --- | --- | --- | --- |
| **Lab** | **GenBank** |  |  |  |  |  |  |
| Ttr_1* | Ttr_GI1 | Galápagos Islands | Skin | 2005 | F | 7-mtDNA regions | b, c, d, e |
| Ttr_2 | Ttr_SA1 | Salinas, Punta Carnero | Skin | 2006 | - | 7-mtDNA regions | All |
| Ttr_3 | Ttr_SA2 | Salinas, Mar Bravo | Skin | 2006 | M | 7-mtDNA regions | All |
| Ttr_4 | Ttr_SA3 | Salinas, Mar Bravo | Skin | 2007 | - | 7-mtDNA regions | All |
| Ttr_5 | Ttr_SA4 | Salinas, Mar Bravo | Skin | 2007 | F | 7-mtDNA regions | All |
| Ttr_6 | Ttr_SA5 | Salinas, Mar Bravo | Skin | 2008 | F | 7-mtDNA regions | All |
| Ttr_7 | Ttr_SA6 | Salinas, Mar Bravo | Skin | 2009 | - | 7-mtDNA regions | All |
| Ttr_8 | Ttr_SA7 | Salinas, Mar Bravo | Skin | 2010 | F | 7-mtDNA regions | All |
| Ttr_9 | Ttr_GG32 | Puná Island | Right mandible | 1992 | - | mtDNA CR: KU992117 | b |
| Ttr_10 | Ttr_GG33 | Jambelí Island | Occipital condyle | 1990 | - | mtDNA CR: KU992120^+^ | a, b, c, d |
| Ttr_11 | Ttr_GG34 | Puná Island | Right mandible | 1991 | - | - |  |
| Ttr_12 | Ttr_GG35 | Puná Island | Right mandible | 1994 | - | *Cytb*: KU992261^+^ |  |
| Ttr_13 | Ttr_UN1 | NA | Occipital condyle | 1995 | - | - |  |
| Ttr_14 | Ttr_UN2 | NA | Right mandible | 1994 | - | - |  |
| Ttr_15 | Ttr_GG36 | Puná Island | Occipital condyle | 1991 | - | - |  |
| Ttr_16 | Ttr_GG37 | Puná Island | Right mandible | 1993 | - | *16S*: KU992038^+^  *Cytb*: KU992262^+^  mtDNA CR: KU992136 | a, b, c, d |
| Ttr_17 | Ttr_GV1 | General Villamil, Playas | Right mandible | 1995 | - | *16S*: KU992039^+^  *Cytb*: KU992263^+^  mtDNA CR: KU992121 | a, b, c, d |
| Ttr_18 | Ttr_ SA8 | Salinas, Punta Carnero | Occipital condyle | 2005 | - | - |  |
| Ttr_19 | Ttr_GG38 | Puná Island | Right mandible | 1994 | - | mtDNA CR: KU992118 | b |
| Ttr_20 | Ttr_GG39 | Puná Island | Occipital condyle | 1990 | - | mtDNA CR: KU992137 | a, b, c, d |
| Ttr_21 | Ttr_GG40 | Puná Island | Occipital condyle | 1991 | - | mtDNA CR: KU992122 | a, b, c, d |

Continuation...

| **Sample ID** | | **Sampling location** | **Tissue type** | **Collection date** | **Molecular sex identification** | **Genes/regions amplified** | **Specific test** |
| --- | --- | --- | --- | --- | --- | --- | --- |
| **Lab** | **GenBank** |  |  |  |  |  |  |
| Ttr_22 | Ttr_UN3 | NA | Right mandible | 1993 | - | 16S: KU992040^+^  *Cytb*: KU992264^+^  mtDNA CR: KU992123 | b, c |
| Ttr_23 | Ttr_GG41 | Puná Island | Occipital condyle | 1991 | - | mtDNA CR: KU992124 | a, b, c, d |
| Ttr_24 | Ttr_GG42 | NA | Occipital condyle | 1992 | - | - |  |
| Ttr_25 | Ttr_GG43 | Puná Island | Occipital condyle | 1994 | - | - |  |
| Ttr_26 | Ttr_UN4 | NA | Right mandible | 1995 | - | 16S: KU992041^+^  *Cytb*: KU992265^+^  mtDNA CR: KU992125 | b, c |
| Ttr_27 | Ttr_GI2 | Galápagos Islands | Right mandible | 2001 | - | - |  |
| Ttr_28 | Ttr_UN5 | NA | Right mandible | 1992 | - | *Cytb*: KU992266^+^  mtDNA CR: KU992126 | b, c |
| Ttr_29 | Ttr_UN6 | NA | Occipital condyle | 1994 | - | *16S*: KU992042^+^  mtDNA CR: KU992119^+^ |  |
| Ttr_30 | Ttr_UN7 | NA | Occipital condyle | 1992 | - | - |  |
| Ttr_31 | Ttr_GG44 | Puná Island | Left mandible | 1992 | - | - |  |
| Ttr_32 | Ttr_SA9 | Salinas, Mar Bravo | Right mandible | 1995 | - | - |  |
| Ttr_33 | Ttr_GG45 | Jambelí Island | Occipital condyle | 1990 | - | mtDNA CR: KU992138 | a, b, c, d |
| Ttr_34 | Ttr_SA10 | Salinas, Mar Bravo | Right mandible | 1997 | - | *16S*: KU992043^+^  *Cytb*: KU992267^+^  mtDNA CR: KU992127 | a, b, c, d |
| Ttr_35 | Ttr_SA11 | Salinas, Mar Bravo | Right mandible | 1995 | - | mtDNA CR: KU992139 | a, b, c, d |
| Ttr_36 | Ttr_SA12 | Salinas, Mar Bravo | Right mandible | 1993 | - | mtDNA CR: KU992140 | a, b, c, d |

Continuation…

| **Sample ID** | | **Sampling location** | **Tissue type** | **Collection date** | **Molecular sex identification** | **Genes/regions amplified** | **Specific test** |
| --- | --- | --- | --- | --- | --- | --- | --- |
| **Lab** | **GenBank** |  |  |  |  |  |  |
| Ttr_37 | Ttr_SA13 | Salinas, Mar Bravo | Right mandible | 2009 | - | *16S*: KU992039^+^  *Cytb*: KU992263^+^  mtDNA CR: KU992121 | a, b, c, d |
| Ttr_38 | Ttr_GG1 | Posorja | Skin | 2013 | M | 7-mtDNA regions | All |
| Ttr_39 | Ttr_GG2 | Posorja | Skin | 2013 | M | 7-mtDNA regions | All |
| Ttr_40 | Ttr_GG3 | Posorja | Skin | 2013 | M | 7-mtDNA regions | All |
| Ttr_41 | Ttr_GG4 | Posorja | Skin | 2013 | F | 7-mtDNA regions | All |
| Ttr_42 | Ttr_GG5 | Posorja | Skin | 2013 | M | 7-mtDNA regions | All |
| Ttr_43 | Ttr_GG6 | Posorja | Skin | 2013 | F | 7-mtDNA regions | All |
| Ttr_44 | Ttr_GG7 | Posorja | Skin | 2013 | M | 7-mtDNA regions | All |
| Ttr_45 | Ttr_GG8 | Posorja | Skin | 2013 | F | 7-mtDNA regions | All |
| Ttr_46 | Ttr_GG9 | Posorja | Skin | 2013 | M | 7-mtDNA regions | All |
| Ttr_47 | Ttr_GG10 | El Morro | Skin | 2013 | F | 7-mtDNA regions | All |
| Ttr_48 | Ttr_GG11 | El Morro | Skin | 2013 | F | 7-mtDNA regions | All |
| Ttr_49 | Ttr_GG12 | Posorja | Skin | 2013 | M | 7-mtDNA regions | All |
| Ttr_50 | Ttr_GG13 | Posorja | Skin | 2013 | M | 7-mtDNA regions | All |
| Ttr_51 | Ttr_GG14 | Posorja | Skin | 2013 | M | 7-mtDNA regions | All |
| Ttr_52 | Ttr_GG15 | Posorja | Skin | 2013 | F | 7-mtDNA regions | All |
| Ttr_53 | Ttr_GG16 | Puná Island | Skin | 2013 | M | 7-mtDNA regions | All |
| Ttr_54 | Ttr_GG17 | Puná Island | Skin | 2013 | M | 7-mtDNA regions | All |
| Ttr_55 | Ttr_GG18 | Posorja | Skin | 2013 | M | 7-mtDNA regions | All |
| Ttr_56 | Ttr_GG19 | Posorja | Skin | 2013 | F | 7-mtDNA regions | All |
| Ttr_57 | Ttr_GG20 | Posorja | Skin | 2013 | F | 7-mtDNA regions | All |
| Ttr_58 | Ttr_GG21 | El Morro | Skin | 2013 | M | 7-mtDNA regions | All |
| Ttr_59 | Ttr_GG22 | El Morro | Skin | 2013 | M | 7-mtDNA regions | All |

Continuation…

| **Sample ID** | | **Sampling location** | **Tissue type** | **Collection date** | **Molecular sex identification** | **Genes/regions amplified** | **Specific test** |
| --- | --- | --- | --- | --- | --- | --- | --- |
| **Lab** | **GenBank** |  |  |  |  |  |  |
| Ttr_60 | Ttr_GG23 | El Morro | Skin | 2013 | M | 7-mtDNA regions | All |
| Ttr_61 | Ttr_GG24 | El Morro | Skin | 2013 | M | 7-mtDNA regions | All |
| Ttr_62 | Ttr_GG25 | El Morro | Skin | 2013 | M | 7-mtDNA regions | All |
| Ttr_63 | Ttr_GG26 | Posorja | Skin | 2013 | M | 7-mtDNA regions | All |
| Ttr_64 | Ttr_GG27 | Posorja | Skin | 2013 | M | 7-mtDNA regions | All |
| Ttr_65 | Ttr_GG28 | Posorja | Skin | 2013 | M | 7-mtDNA regions | All |
| Ttr_66 | Ttr_GG29 | Posorja | Skin | 2013 | M | 7-mtDNA regions | All |
| Ttr_67 | Ttr_GG30 | Posorja | Skin | 2013 | F | 7-mtDNA regions | All |
| Ttr_68 | Ttr_GG31 | Posorja | Skin | 2013 | M | 7-mtDNA regions | All |
| Ttr_69 | Ttr_PE1 | Peru: Santa Rosa | Skin | 2013 | NA | 7-mtDNA regions | b, c, d, e |
