# Supplementary material for: Genetic divergence and fine scale population structure of the common bottlenose dolphin (Tursiops truncatus, Montagu) found in the Gulf of Guayaquil, Ecuador
Source: PeerJ. 2018 Apr 9;6:e4589. doi: 10.7717/peerj.4589 (PMC5916226; doi:10.7717/peerj.4589)
Supplement: Supplemental Information 4 — Acronyms: bp: base pair, Ta: Temperature annealing, mM: milimolar, CR: control region, COI: cytochrome oxidase I, COII: cytochrome oxidase II, Cyt b: cytochrome b, ND1-ND2: NADH dehydrogenase subunit I-II. [file peerj-06-4589-s004.docx]

| **Primer** | **Sequence** | **Size (bp)** | **mtDNA region** | **Ta (˚C)** | **MgCl_2_ (mM)** | **References** |
| --- | --- | --- | --- | --- | --- | --- |
| mt12F-12S RNA | TTA CAC ATG CAA GCA TCC GC | ~ 1,062 | *12S rRNA-16S rRNA* | 55 | 1.5 | Cunha et al., 2011 |
| mt12R-12S RNA | GGT ACT CTC TCT ATA GCG CC |  |  |  |  |  |
| 16SarL | CGC CTG TTT ATC AAA AAC AT | ~ 590 | *16S rRNA* | 55 | 1.5 | Palumbi et al., 1991 |
| 16SbrH | CCG GTC TGA ACT CAG ATC ACG T |  |  |  |  |  |
| ND1F | TCA GAA CTC GTA TCT GGC | ~ 956 | *ND1-ND2* | 51 | 1.5 | Xiong et al., 2009 |
| ND1R | ATT AGT CCT GTG CTT AGG G |  |  |  |  |  |
| COX1F | TGC CTA CTC GGC CAT TTT AC | ~ 756 | *COI* | 52 | 1.5 | Amaral et al., 2007 |
| COX1R | TGA AAC CCA GGA AGC CAA TA |  |  |  |  |  |
| CO2LCet | TAA ART CTT ACA TAA CTT TGT C | ~ 684 | *COII* | 50 | 2.0 | McGowen et al., 2008 |
| CO2RCet | TCT CAA TCT TTA ACT TAA AAG G |  |  |  |  |  |
| L14724 | TGA CTT GAA RAA CCA YCG TTG | ~ 465 | *Cytb* | 48 | 1.5 | Palumbi et al., 1991 |
| H15149 | CAG AAT GAT ATT TGT CCT CA |  |  |  |  | Kocher et al., 1989 |
| dLp1.5t-pro | TCA CCC AAA GCT GRA RTT CTA |  | CR | 55 | 1.5 | Dalebout et al., 1998 |
| dLp8G | GGA GTA CTA TGT CCT GTA ACC A | ~ 800 |  |  |  | Lento et al., 1997 |
| dlp5 | CCA TCG WGA TGT CTT ATT TAA GRG GAA | ~ 550 |  |  |  | Dalebout et al., 1998 |
| dlp4 | CGG GTT GCT GGT TTC ACG | ~ 400 |  |  |  | Pichler & Baker, 2000 |

**References**

**Amaral AR, Sequeira M, Coelho MM. 2007.** A first approach to the usefulness of cytochrome c oxidase I barcodes in the identification of closely related delphinid cetacean species. *Marine and Freshwater Research* **58(6)**:505–510. DOI: 10.1071/MF07050.

**Cunha HA, Moraes LC, Medeiros BV, Lailson-Brito J, da Silva VM, Solé-Cava MA, Schrago CG*.* 2011.** Phylogenetic status and timescale for the diversification of *Steno* and *Sotalia* dolphins. *PLoS ONE.* **6(12)**:e28297. DOI: [10.1371/journal.pone.0028297](https://doi.org/10.1371/journal.pone.0028297).

**Dalebout ML, Van Helden A, Van Waerebeek K, Baker CS. 1998.** Molecular genetic identification of southern hemisphere beaked whales (Cetacea: Ziphiidae). *Molecular Ecology* **7(6)**:687–695. DOI: 10.1046/j.1365-294x.1998.00380.x.

**Kocher TD, Thomas WK, Meyer A, Edwards SV, Pããbo S, Villablanca FX, Wilson AC. 1989.** Dynamics of mitochondrial DNA evolution in animals: amplification and sequencing with conserved primers. *Proceedings of the National Academy of Sciences of the United States of America* **86(16)**:6196–6200.

**Lento GM, Patenaude NJ, Baker CS. 1997.** Molecular genetic identification of whale and dolphin products for sale in Japan and Korea, 1995-97. Report to the Scientific Committee of the International Whaling Commission. SC/49/O21.

**McGowen MR, Clark C, Gatesy J. 2008.** The vestigial olfactory receptor subgenome of odontocete whales: phylogenetic congruence between gene-tree reconciliation and supermatrix methods. *Systematic* *Biology* **57(4)**:574–590. DOI: 10.1080/10635150802304787.

**Palumbi SR, Martin A, Romano S, McMillan WO, Stice L, Grabowsky G. 1991.** The simple fool's guide to PCR. Honolulu: University of Hawaii.

**Pichler FB, & Baker CS. 2000.** Loss of diversity in the endemic Hector's dolphin due to fisheries-related mortality. *Proceedings of the Royal Society of London B: Biological Sciences* **267(1438)**:97–102. DOI: 10.1098/rspb.2000.0972.

**Xiong Y, Brandley MC, Xu S, Zhou K, Yang G. 2009.** Seven new dolphin mitochondrial genomes and a time-calibrated phylogeny of whales. *BMC* *Evolutionary Biology* **9(1)**:1–13. DOI: 10.1186/1471-2148-9-20.
