# Supplementary material for: Genetic divergence and fine scale population structure of the common bottlenose dolphin (Tursiops truncatus, Montagu) found in the Gulf of Guayaquil, Ecuador
Source: PeerJ. 2018 Apr 9;6:e4589. doi: 10.7717/peerj.4589 (PMC5916226; doi:10.7717/peerj.4589)
Supplement: Supplemental Information 5 — The table includes the accession numbers and sequences geographic location. Number of samples by haplotype (n). Acronyms: Hap: Haplotype, C: coastal, O: Offshore, P: pelagic, U: unknown. [file peerj-06-4589-s005.docx]

| **Haplotype** | **n** | **Geographic location** | **Ecotype** | **Accession Number** |
| --- | --- | --- | --- | --- |
| **Pacific Ocean Basin** | | | | |
| Hap 15 | 1 | Gulf of California | C/O | DQ105702.1 |
| Hap 16 | 2 | Gulf of California | C, O | DQ105703.1, HQ206663.1 |
| Hap 17 | 2 | Gulf of California | O | DQ105704.1, HQ206678.1 |
| Hap 18 | 1 | Gulf of California | C/O | DQ105705.1 |
| Hap 19 | 1 | Gulf of California | C | DQ105706.1 |
| Hap 20 | 1 | Gulf of California | O | DQ105707.1 |
| Hap 21 | 2 | Gulf of California | C, O | DQ105708.1, HQ206666.1 |
| Hap 22 | 2 | Gulf of California | C | DQ105709.1, HQ206669.1 |
| Hap 23 | 3 | Gulf of California | O | DQ105710.1, HE617285.1, DQ206702.1 |
| Hap 24 | 1 | Gulf of California | C, O | DQ105711.1 |
| Hap 25 | 3 | Gulf of California, Hawaiian Islands | O | DQ105712.1, HQ206681.1, EF672716.1 |
| Hap 26 | 1 | Gulf of California | O | DQ105713.1 |
| Hap 27 | 1 | Gulf of California | O | DQ105714.1 |
| Hap 28 | 2 | Gulf of California | C, O | DQ105715.1, HE617270.1 |
| Hap 29 | 3 | Gulf of California, Hawaiian Islands | C, P | DQ105717.1, HQ206660.1, EF672706.1 |
| Hap 30 | 1 | Gulf of California | C | DQ105719.1 |
| Hap 31 | 1 | Gulf of California | U | DQ105720.1 |
| Hap 32 | 1 | Gulf of California | U | DQ105722.1 |
| Hap 33 | 2 | Gulf of California | O | DQ105723.1 |
| Hap 34 | 2 | Gulf of California | O | DQ105724.1 |
| Hap 35 | 1 | Gulf of California | O | DQ105725.1 |
| Hap 36 | 2 | Gulf of California | O | DQ105727.1 |
| Hap 37 | 1 | Gulf of California | O | DQ105730.1 |
| Hap 38 | 1 | Gulf of California | U | DQ105731.1 |
| Hap 39 | 2 | Gulf of California | C | DQ105732.1 |
| Hap 40 | 2 | Gulf of California | O | DQ105733.1 |
| Hap 41 | 1 | Gulf of California | O | HE617259.1 |
| Hap 42 | 1 | Gulf of California | U | HE617262.1 |
| Hap 43 | 2 | Gulf of California | C | HE617263.1 |
| Hap 44 | 2 | Gulf of California | C/O | HE617264.1 |
| Hap 45 | 2 | Gulf of California | C | HE617265.1 |
| Hap 46 | 3 | Gulf of California | C | HE617267.1 |
| Hap 47 | 2 | Gulf of California | O | HE617268.1 |
| Hap 48 | 1 | Gulf of California | U | HE617269.1 |
| Hap 49 | 1 | Gulf of California | U | HE617271.1 |

**Continue…**

| **Haplotype** | **n** | **Geographic location** | **Ecotype** | **Accession Number** |
| --- | --- | --- | --- | --- |
| **Pacific Ocean Basin** | | | | |
| Hap 50 | 2 | Gulf of California | O | HE617274.1 |
| Hap 51 | 1 | Gulf of California | U | HE617275.1 |
| Hap 52 | 1 | Gulf of California | U | HE617276.1 |
| Hap 53 | 1 | Gulf of California | O | HE617277.1 |
| Hap 54 | 1 | Gulf of California | O | HE617278.1 |
| Hap 55 | 1 | Gulf of California | U | HE617284.1 |
| Hap 56 | 1 | Gulf of California | U | HE617286.1 |
| Hap 57 | 1 | Gulf of California | O | HE617287.1 |
| Hap 58 | 1 | Gulf of California | O | HE617289.1 |
| Hap 59 | 1 | Gulf of California | O | HE617290.1 |
| Hap 60 | 1 | Gulf of California | O | HE617291.1 |
| Hap 61 | 1 | Gulf of California | O | HE617292.1 |
| Hap 62 | 1 | Gulf of California | O | HE617293.1 |
| Hap 63 | 2 | Gulf of California | C | HE617294.1, HQ206679.1 |
| Hap 64 | 1 | Gulf of California | O | HQ206670.1 |
| Hap 65 | 1 | Gulf of California | O | HQ206673.1 |
| Hap 66 | 2 | Gulf of California, Japan | O | HQ206674.1, AB303162.1 |
| Hap 67 | 1 | Gulf of California | O | HQ206675.1 |
| Hap 68 | 1 | Gulf of California | O | HQ206676.1 |
| Hap 69 | 1 | Gulf of California | C/O | HQ206677.1 |
| Hap 70 | 1 | Gulf of California | O | HQ206682.1 |
| Hap 71 | 1 | Gulf of California | U | HQ206684.1 |
| Hap 72 | 1 | Gulf of California | O | HQ206685.1 |
| Hap 73 | 1 | Gulf of California | O | HQ206686.1 |
| Hap 74 | 1 | Gulf of California | O | HQ206687.1 |
| Hap 75 | 1 | Gulf of California | U | HQ206688.1 |
| Hap 76 | 1 | Gulf of California | U | HQ206690.1 |
| Hap 77 | 1 | Gulf of California | U | HQ206691.1 |
| Hap 78 | 1 | Gulf of California | U | HQ206692.1 |
| Hap 79 | 1 | Gulf of California | U | HQ206693.1 |
| Hap 80 | 1 | Gulf of California | O | HQ206697.1 |
| Hap 81 | 1 | Gulf of California | U | HQ206698.1 |
| Hap 82 | 1 | Gulf of California | O | HQ206699.1 |
| Hap 83 | 1 | Gulf of California | U | HQ206700.1 |
| Hap 84 | 1 | Gulf of California | U | HQ206701.1 |

**Continue...**

| **Haplotype** | **n** | **Geographic location** | **Ecotype** | **Accession Number** |
| --- | --- | --- | --- | --- |
| **Pacific Ocean Basin** | | | | |
| Hap 85 | 1 | Gulf of California | O | HQ206703.1 |
| Hap 86 | 1 | Gulf of California | U | HQ206704.1 |
| Hap 87 | 1 | Gulf of California | O | HQ206709.1 |
| Hap 88 | 1 | Gulf of California | O | HQ206714.1 |
| Hap 89 | 1 | Hawaiian Islands | P | EF672700.1 |
| Hap 90 | 1 | Hawaiian Islands | P | EF672701.1 |
| Hap 91 | 2 | Hawaiian Islands | P | EF672702.1, NC_01259.1 |
| Hap 92 | 1 | Hawaiian Islands | P | EF672703.1 |
| Hap 93 | 1 | Hawaiian Islands | P | EF672704.1 |
| Hap 94 | 1 | Hawaiian Islands | P | EF672705.1 |
| Hap 95 | 3 | Hawaiian Islands, China, Japan | P | EF672707.1, AF355585.1, AB303156.1 |
| Hap 96 | 1 | Hawaiian Islands | P | EF672708.1 |
| Hap 97 | 1 | Hawaiian Islands | P | EF672709.1 |
| Hap 98 | 1 | Hawaiian Islands | P | EF672710.1 |
| Hap 99 | 1 | Hawaiian Islands | P | EF672711.1 |
| Hap 100 | 9 | Hawaiian Islands, China, Japan | P, U | EF672712.1, AF459509.1, AF459510.1, AF459511.1, AF459512.1, AF459513.1, AF459514.1, AF459515.1, AB303159.1 |
| Hap 101 | 1 | Hawaiian Islands | P | EF672714.1 |
| Hap 102 | 1 | Hawaiian Islands | P | EF672715.1 |
| Hap 103 | 1 | Hawaiian Islands | P | EF672717.1 |
| Hap 104 | 1 | Hawaiian Islands | P | EF672718.1 |
| Hap 105 | 1 | Hawaiian Islands | P | EF672719.1 |
| Hap 106 | 1 | Hawaiian Islands | P | EF672721.1 |
| Hap 107 | 1 | Hawaiian Islands | P | EF672723.1 |
| Hap 108 | 1 | Hawaiian Islands | P | EF672724.1 |
| Hap 109 | 2 | China | U | AF355586.1, AF459508.1 |
| Hap 110 | 1 | China | U | AF355587.1 |
| Hap 111 | 1 | China | U | AF355584.1 |
| Hap 112 | 1 | China | U | AF355583.1 |
| Hap 113 | 1 | China | U | AF459522.1 |
| Hap 114 | 1 | China | U | AF459523.1 |
| Hap 115 | 2 | Japan | U | AB610376.1, AB303163.1 |
| Hap 116 | 1 | Japan | U | AB303154.1 |
| Hap 117 | 1 | Japan | U | AB303155.1 |

**Continue…**

| **Haplotype** | **n** | **Geographic location** | **Ecotype** | **Accession Number** |
| --- | --- | --- | --- | --- |
| **Pacific Ocean Basin** | | | | |
| Hap 118 | 1 | Japan | U | AB303157.1 |
| Hap 119 | 1 | Japan | U | AB303158.1 |
| Hap 120 | 1 | Japan | U | AB303160.1 |
| Hap 121 | 1 | Japan | U | AB303164.1 |
| Hap 122 | 1 | Japan | U | AB303166.1 |
| Hap 123 | 1 | Japan | U | AB303167.1 |
| Hap 124 | 1 | Japan | U | AB303168.1 |
| Hap 125 | 1 | Japan | U | AB303169.1 |
| Hap 126 | 1 | Japan | U | AB303170.1 |
| Hap 127 | 1 | Japan | U | AB303171.1 |
| Hap 128 | 1 | Japan | U | AB303172.1 |
| Hap 129 | 1 | Japan | U | AB303173.1 |
| Hap 130 | 1 | Japan | U | AB303174.1 |
| Hap 131 | 1 | New Zealand | P | EU121118.1 |
| **Atlantic Ocean Basin, Mediterranean Sea, Black Sea** | | | | |
| Hap 132 | 2 | Eastern Mediterranean | C | KF570316.1, KF570318.1 |
| Hap 133 | 3 | Eastern Mediterranean  Scotland | C | KF570317.1, KF570347.1, KF570351.1 |
| Hap 134 | 2 | Eastern Mediterranean | C | KF570319.1 |
| Hap 135 | 1 | Eastern Mediterranean | C | KF570320.1 |
| Hap 136 | 17 | Eastern Mediterranean,  Eastern North Atlantic,  Black Sea  West Mediterranean,  Madeira, Portugal, Ireland, France | C, P | KF570321.1, KT601195.1, KT601200.1, AY963588.1, AY963599.1, AY963611.1, DQ525365.1, DQ525374.1, DQ073720.1, DQ073722.1, DQ073727.1, HQ634249.1, KF281706.1 |
| Hap 137 | 4 | Eastern Mediterranean  Portugal | C, P | KF570322.1, AY963600.1, AYA93603.1, DQ073723.1 |
| Hap 138 | 1 | Eastern Mediterranean | C | KF570323.1 |
| Hap 139 | 2 | Eastern Mediterranean  Black Sea | C | KF570324.1, KF570334.1 |
| Hap 140 | 2 | Black Sea | C | KF570326.1, AY963590.1 |
| Hap 141 | 3 | Black Sea | C | KF570327.1, KF570331.1, AY963591.1 |
| Hap 142 | 1 | Black Sea | C | KF570328.1 |
| Hap 143 | 1 | Black Sea | C | KF570329.1 |
| Hap 144 | 2 | Black Sea | C | KF570332.1, AY963592.1 |
| Hap 145 | 1 | Black Sea | C | KF570333.1 |

**Continue…**

| **Haplotype** | **n** | **Geographic location** | **Ecotype** | **Accession Number** |
| --- | --- | --- | --- | --- |
| **Atlantic Ocean Basin, Mediterranean Sea, Black Sea** | | | | |
| Hap 146 | 11 | Scotland, Eastern North Atlantic, Portugal, Ireland, United Kingdom | C, P | KF570345.1, KF570346.1, KF570348.1, KF570349.1, KF570350.1, KT601190.1, KT601191.1, AY963622.1, DQ073726.1, HQ634245.1, AF268357.1 |
| Hap 147 | 7 | Scotland | C | KF570352.1 |
| Hap 148 | 1 | Western North Atlantic | C | KF570370.1 |
| Hap 149 | 1 | Western North Atlantic | C | KF570371.1 |
| Hap 150 | 1 | Western North Atlantic | C | KF570372.1 |
| Hap 151 | 2 | Western North Atlantic | C | KF570373.1, KF570374.1 |
| Hap 152 | 1 | Western North Atlantic | C | KF570375.1 |
| Hap 153 | 1 | Western North Atlantic | C | KF570376.1 |
| Hap 154 | 1 | Western North Atlantic | C | KF570378.1 |
| Hap 155 | 2 | Western North Atlantic | P | KF570379.1, DQ073655.1 |
| Hap 156 | 1 | Western North Atlantic | P | KF570381.1 |
| Hap 157 | 1 | Western North Atlantic | P | KF570383.1 |
| Hap 158 | 1 | Western North Atlantic | P | KF570384.1 |
| Hap 159 | 16 | Western North Atlantic  Eastern North Atlantic, Azores, Madeira, Portugal, Ireland | P, C | KF570385.1, KT601204.1, AY963624.1, DQ525357.1, DQ525362.1, DQ525387.1, DQ073645.1, DQ073651.1, DQ073676.1, DQ073689.1, DQ073694.1, DQ073697.1, DQ073703.1, DQ073708.1, GQ241419.1, HQ634250.1 |
| Hap 160 | 1 | Western North Atlantic | P | KF570386.1 |
| Hap 161 | 1 | Western North Atlantic | P | KF570387.1 |
| Hap 162 | 2 | Eastern North Atlantic, Ireland | C | KT601188.1, HQ634246.1 |
| Hap 163 | 2 | Eastern North Atlantic, Ireland | C | KT601189.1, HQ634251.1 |
| Hap 164 | 3 | Eastern North Atlantic | C | KT601194.1, KT601205.1, AY963619.1 |
| Hap 165 | 6 | Eastern North Atlantic, Azores, Portugal, Ireland | C | KT601196.1, KT601206.1, AY963623.1, DQ525363.1, DQ073692.1, HQ634255.1 |
| Hap 166 | 1 | Eastern North Atlantic | P | KT601197.1 |
| Hap 167 | 1 | Eastern North Atlantic | P | KT601198.1 |

**Continue…**

| **Haplotype** | **n** | **Geographic location** | **Ecotype** | **Accession Number** |
| --- | --- | --- | --- | --- |
| **Atlantic Ocean Basin, Mediterranean Sea, Black Sea** | | | | |
| Hap 168 | 7 | Eastern North Atlantic, Ireland | P | KT601199.1, KT601203.1, DQ525364.1, DQ073675.1, DQ073716.1, HQ634247.1 |
| Hap 169 | 1 | Eastern North Atlantic | P | KT601202.1 |
| Hap 170 | 1 | Eastern North Atlantic, | P | KT601207.1 |
| Hap 171 | 1 | Black Sea | C | AY963589.1 |
| Hap 172 | 1 | Black Sea | C | AY963593.1 |
| Hap 173 | 1 | East Mediterranean | C | AY963594.1 |
| Hap 174 | 1 | East Mediterranean | C | AY963595.1 |
| Hap 175 | 1 | East Mediterranean | C | AY963596.1 |
| Hap 176 | 1 | East Mediterranean | C | AY963598.1 |
| Hap 177 | 1 | East Mediterranean | C | AY963601.1 |
| Hap 178 | 3 | East Mediterranean  Azores | C, P | AY963604.1, AY963609.1, DQ073711.1 |
| Hap 179 | 1 | West Mediterranean | C | AY963605.1 |
| Hap 180 | 1 | West Mediterranean | C | AY963606.1 |
| Hap 181 | 7 | West Mediterranean, Madeira, Azores, Portugal | C, P | AY963607.1, DQ525366.1, DQ073660.1, DQ073679.1, DQ073680.1, DQ073706.1, DQ073721.1 |
| Hap 182 | 1 | West Mediterranean | C | AY963608.1 |
| Hap 183 | 1 | West Mediterranean | C | AY963610.1 |
| Hap 184 | 1 | West Mediterranean | C | AY963612.1 |
| Hap 185 | 1 | West Mediterranean | C | AY963613.1 |
| Hap 186 | 1 | West Mediterranean | C | AY963614.1 |
| Hap 187 | 1 | West Mediterranean | C | AY963615.1 |
| Hap 188 | 3 | West Mediterranean, Madeira, Ireland | C, P | AY963616.1, DQ525371.1, HQ634254.1 |
| Hap 189 | 1 | Eastern North Atlantic | C | AY963617.1 |
| Hap 190 | 3 | Eastern North Atlantic  Portugal | C, P | AY963618.1, DQ073718.1, DQ073728.1 |
| Hap 191 | 2 | Eastern North Atlantic  Portugal | C, P | AY963620.1, DQ073724.1 |
| Hap 192 | 1 | Eastern North Atlantic | C | AY963621.1 |
| Hap 193 | 1 | Eastern North Atlantic | C | AY963626.1 |
| Hap 194 | 4 | Azores | P | DQ525358.1, DQ073656.1, DQ073674.1, DQ073690.1 |
| Hap 195 | 3 | Portugal | P | DQ525360.1, DQ073678.1, DQ073729.1 |

**Continue…**

| **Haplotype** | **n** | **Geographic location** | **Ecotype** | **Accession Number** |
| --- | --- | --- | --- | --- |
| **Atlantic Ocean Basin, Mediterranean Sea, Black Sea** | | | | |
| Hap 196 | 2 | Azores | P | DQ525361.1, DQ073648.1 |
| Hap 197 | 4 | Madeira, Azores | P | DQ525367.1, DQ525376.1, DQ525380.1, DQ073653.1 |
| Hap 198 | 2 | Madeira, Azores | P | DQ525369.1, DQ073671.1 |
| Hap 199 | 3 | Madeira, Azores | P | DQ525370.1, DQ073683.1, DQ073686.1 |
| Hap 200 | 8 | Madeira, Portugal, Azores | P | DQ525373.1, DQ525379.1, DQ525382.1, DQ525383.1, DQ525384.1, DQ073654.1, DQ073672.1, DQ073712.1 |
| Hap 201 | 1 | Madeira | P | DQ525377.1 |
| Hap 202 | 1 | Madeira | P | DQ525378.1 |
| Hap 203 | 7 | Madeira, Azores | P | DQ525381.1, DQ525388.1, DQ073657.1, DQ073664.1, DQ073665.1, DQ073666.1, DQ073713.1 |
| Hap 204 | 2 | Madeira, Azores | P | DQ525386.1, DQ073668.1 |
| Hap 205 | 3 | Madeira, Azores | P | DQ073641.1, DQ073682.1, DQ073704.1 |
| Hap 206 | 5 | Azores | P | DQ073642.1, DQ073658.1, DQ073667.1, DQ073677.1, DQ073714.1 |
| Hap 207 | 5 | Azores | P | DQ073646.1, DQ073647.1, DQ073662.1, DQ073685.1, DQ073717.1 |
| Hap 208 | 1 | Azores | P | DQ073649.1 |
| Hap 209 | 1 | Azores | P | DQ073650.1 |
| Hap 210 | 3 | Azores | P | DQ073652.1, DQ073695.1, DQ073702.1 |
| Hap 211 | 2 | Azores | P | DQ073663.1, DQ073696.1 |
| Hap 212 | 1 | Azores | P | DQ073681.1 |
| Hap 213 | 1 | Azores | P | DQ073688.1 |
| Hap 214 | 1 | Azores | P | DQ073693.1 |
| Hap 215 | 2 | Azores | P | DQ073698.1, DQ073700.1 |
| Hap 216 | 1 | Azores | P | DQ073701.1 |
| Hap 217 | 1 | Azores | P | DQ073705.1 |
| Hap 218 | 1 | Azores | P | DQ073707.1 |
| Hap 219 | 1 | Azores | P | DQ073709.1 |
| Hap 220 | 1 | Azores | P | DQ073710.1 |
| Hap 221 | 1 | Ireland | C | HQ634257.1 |

**References**

**Ji G, Yang G, Liu S, Shou K.** A study on the variability of the mitochondrial DNA control region of bottlenose dolphins (genus: *Tursiops*) in Chinese waters. Unpublished.

**Kita YF, Hosomichi K, Suzuki S, Inoko H, Shiina T, Watanabe M, Tanaka A, Horie T, Ohizumi H, Tanaka S, Iwasaki T, Ota M, Kulski J. 2013.** Genetic and family structure in a group of 165 common bottlenose dolphins caught off the Japanese coast. *Marine Mammal Science* **29(3)**:474–496. DOI: 10.1111/j.1748-7692.2012.00581.x.

**Martien K, Robertson K.** Population structure of island-associated dolphins II: using mitochondrial and microsatellite markers of common bottlenose dolphins around the main Hawaiian Islands. Unpublished.

**Moura AE, Nielsen SC, Vilstrup JT, Moreno-Mayar JV, Gilbert MT, Gray HW, Natoli, A, Möller, L, Hoelzel AR*.* 2013.** Recent diversification of a marine genus (*Tursiops* spp.) tracks habitat preference and environmental change. *Systematic Biology* **62(6)**:865–877. DOI: 10.1093/sysbio/syt051.

**Nykanen M, Foote A.D.** Reconstructing the post-glacial colonization of the northern extreme of the range of a top marine predator, the bottlenose dolphin. Unpublished.

**Natoli A, Peddemors VM, Hoelzel AR. 2004.** Population structure and speciation in the genus *Tursiops* based on microsatellite and mitochondrial DNA analyses. *Journal of Evolutionary Biology* **17(2)**:363–375. DOI: 10.1046/j.1420-9101.2003.00672.x.

**Perrin WF, Thieleking JL, Walter WA, Archer FI, Robertson KM. 2011.** Common bottlenose dolphins (*Tursiops truncatus*) in California waters: cranial differentiation of coastal and offshore ecotypes. *Marine Mammal Science* **27(4)**:769–792. DOI: 10.1111/j.1748-7692.2010.00442.x.

**Quérouil S, Silva MA, Freitas L, Prieto R, Magalhães S, Dinis A, Alves F, Matos JA, Mendoça D, Hammond PS, Santos RS*.* 2007.** High gene flow in oceanic bottlenose dolphins (*Tursiops truncatus*) of the North Atlantic. *Conservation Genetics* **8(6)**:1405–1419. DOI: 10.1007/s10592-007-9291-5.

**Segura I, Rocha-Olivares A, Flores-Ramirez S, Rojas-Bracho L. 2006.** Conservation implications of the genetic and ecological distinction of *Tursiops* *truncatus* ecotypes in the Gulf of California. *Biological Conservation* **133(3):** 336–346. DOI: 10.1016/j.biocon.2006.06.017.

**Segura I, Rocha-Olivares A, Heckel G, Rojo-Arreola L, Gallo-Reynoso J, Hoelzel R.** Ecological affinity and restricted gene flow of the bottlenose dolphin, *Tursiops* *truncatus*, in the Gulf of California and Pacific Ocean. Unpublished
