# Supplementary material for: Genetic divergence and fine scale population structure of the common bottlenose dolphin (Tursiops truncatus, Montagu) found in the Gulf of Guayaquil, Ecuador
Source: PeerJ. 2018 Apr 9;6:e4589. doi: 10.7717/peerj.4589 (PMC5916226; doi:10.7717/peerj.4589)
Supplement: Supplemental Information 6 — The table includes the accession numbers, haplotype name, sequences geographic location, and ecotypes. Number of samples by haplotype (n). Acronyms: E: Ecotype, C: coastal, P: pelagic, O: offshore, U: unknown. [file peerj-06-4589-s006.docx]

| **Accession number** | **Haplotype** | **Geographical location** | **E** | **Reference** |
| --- | --- | --- | --- | --- |
| **Pacific Ocean basin** | | | | |
| DQ105702.1 | TTGC01 | Gulf of California | C/O | Segura et al., 2006 |
| DQ105703.1 | TTGC02 | Gulf of California | C/O | Segura et al., 2006 |
| DQ105704.1 | TTGC03 | Gulf of California | O | Segura et al., 2006 |
| DQ105705.1 | TTGC04 | Gulf of California | C/O | Segura et al., 2006 |
| DQ105706.1 | TTGC05 | Gulf of California | C | Segura et al., 2006 |
| DQ105707.1 | TTGC06 | Gulf of California | O | Segura et al., 2006 |
| DQ105708.1 | TTGC07 | Gulf of California | C/O | Segura et al., 2006 |
| DQ105709.1 | TTGC08 | Gulf of California | C | Segura et al., 2006 |
| DQ105710.1 | TTGC09 | Gulf of California | O | Segura et al., 2006 |
| DQ105711.1 | TTGC10 | Gulf of California | C/O | Segura et al., 2006 |
| DQ105712.1 | TTGC11 | Gulf of California | O | Segura et al., 2006 |
| DQ105713.1 | TTGC12 | Gulf of California | O | Segura et al., 2006 |
| DQ105714.1 | TTGC13 | Gulf of California | O | Segura et al., 2006 |
| DQ105715.1 | TTGC14 | Gulf of California | C | Segura et al., 2006 |
| DQ105716.1 | TTGC15 | Gulf of California | C | Segura et al., 2006 |
| DQ105717.1 | TTGC16 | Gulf of California | C | Segura et al., 2006 |
| DQ105718.1 | TTGC17 | Gulf of California | O | Segura et al., 2006 |
| DQ105719.1 | TTGC18 | Gulf of California | C | Segura et al., 2006 |
| DQ105720.1 | TTGC19 | Gulf of California | U | Segura et al., 2006 |
| DQ105721.1 | TTGC20 | Gulf of California | U | Segura et al., 2006 |
| DQ105722.1 | TTGC21 | Gulf of California | U | Segura et al., 2006 |
| DQ105723.1 | TTGC22 | Gulf of California | O | Segura et al., 2006 |
| DQ105724.1 | TTGC23 | Gulf of California | O | Segura et al., 2006 |
| DQ105725.1 | TTGC24 | Gulf of California | O | Segura et al., 2006 |
| DQ105726.1 | TTGC25 | Gulf of California | O | Segura et al., 2006 |
| DQ105727.1 | TTGC26 | Gulf of California | O | Segura et al., 2006 |
| DQ105728.1 | TTGC27 | Gulf of California | O | Segura et al., 2006 |

**Continue…**

| **Accession number** | **Haplotype** | **Geographical location** | **Ecotype** | **Reference** |
| --- | --- | --- | --- | --- |
| **Pacific Ocean basin** | | | | |
| DQ105729.1 | TTGC28 | Gulf of California | O | Segura et al., 2006 |
| DQ105730.1 | TTGC29 | Gulf of California | O | Segura et al., 2006 |
| DQ105731.1 | TTGC30 | Gulf of California | U | Segura et al., 2006 |
| KF5700389 | GC1 | Gulf of California | C | Moura et al., 2013 |
| DQ105733.1 | TTGC32 | Gulf of California | O | Segura et al., 2006 |
| HE617258.1 | TTGC38 | Gulf of California | O | Segura et al., 2006 |
| HE617259.1 | TTGC39 | Gulf of California | O | Segura et al., 2006 |
| HE617261.1 | TTGC41 | Gulf of California | U | Segura et al., 2006 |
| HE617262.1 | TTGC42 | Gulf of California | U | Segura et al., 2006 |
| HE617263.1 | TTGC43 | Gulf of California | C | Segura et al., 2006 |
| HE617264.1 | TTGC44 | Gulf of California | C/O | Segura et al., 2006 |
| HE617265.1 | TTGC45 | Gulf of California | C | Segura et al., 2006 |
| HE617266.1 | TTGC46 | Gulf of California | U | Segura et al., 2006 |
| HE617267.1 | TTGC47 | Gulf of California | C | Segura et al., 2006 |
| HE617268.1 | TTGC48 | Gulf of California | O | Segura et al., 2006 |
| HE617269.1 | TTGC49 | Gulf of California | C/O | Segura et al., 2006 |
| HE617271.1 | TTGC51 | Gulf of California | C/O | Segura et al., 2006 |
| HE617272.1 | TTGC52 | Gulf of California | O | Segura et al., 2006 |
| HE617273.1 | TTGC53 | Gulf of California | O | Segura et al., 2006 |
| HE617274.1 | TTGC54 | Gulf of California | O | Segura et al., 2006 |
| HE617275.1 | TTGC55 | Gulf of California | U | Segura et al., 2006 |
| HE617276.1 | TTGC56 | Gulf of California | U | Segura et al., 2006 |
| HE617277.1 | TTGC57 | Gulf of California | O | Segura et al., 2006 |
| HE617278.1 | TTGC58 | Gulf of California | O | Segura et al., 2006 |
| HE617284.1 | TTGC64 | Gulf of California | U | Segura et al., 2006 |
| HE617286.1 | TTGC66 | Gulf of California | U | Segura et al., 2006 |

**Continue…**

| **Accession number** | **Haplotype** | **Geographical location** | **E** | **Reference** |
| --- | --- | --- | --- | --- |
| **Pacific Ocean basin** | | | | |
| HE617287.1 | TTGC67 | Gulf of California | O | Segura et al., unpublished |
| HE617289.1 | TTGC69 | Gulf of California | O | Segura et al., unpublished |
| HE617290.1 | TTGC70 | Gulf of California | O | Segura et al., unpublished |
| HE617291.1 | TTGC71 | Gulf of California | O | Segura et al., unpublished |
| HE617292.1 | TTGC72 | Gulf of California | O | Segura et al., unpublished |
| HE617293.1 | TTGC73 | Gulf of California | O | Segura et al., unpublished |
| HE617294.1 | TTGC74 | Gulf of California | C | Segura et al., unpublished |
| HE617296.1 | TTGC76 | Gulf of California | O | Segura et al., unpublished |
| HE617297.1 | TTGC77 | Gulf of California | O | Segura et al., unpublished |
| HQ206667.1 | CA_Ttru_09 | Gulf of California | O | Perrin et al., 2011 |
| HQ206668.1 | CA_Ttru_10 | Gulf of California | O | Perrin et al., 2011 |
| HQ206670.1 | CA_Ttru_12 | Gulf of California | O | Perrin et al., 2011 |
| HQ206673.1 | CA_Ttru_15 | Gulf of California | O | Perrin et al., 2011 |
| HQ206674.1 | CA_Ttru_16 | Gulf of California | O | Perrin et al., 2011 |
| HQ206675.1 | CA_Ttru_17 | Gulf of California | O | Perrin et al., 2011 |
| HQ206676.1 | CA_Ttru_18 | Gulf of California | O | Perrin et al., 2011 |
| HQ206680.1 | CA_Ttru_22 | Gulf of California | O | Perrin et al., 2011 |
| HQ206682.1 | CA_Ttru_24 | Gulf of California | O | Perrin et al., 2011 |
| HQ206684.1 | CA_Ttru_26 | Gulf of California | U | Perrin et al., 2011 |
| HQ206685.1 | CA_Ttru_27 | Gulf of California | O | Perrin et al., 2011 |
| HQ206686.1 | CA_Ttru_28 | Gulf of California | O | Perrin et al., 2011 |
| HQ206687.1 | CA_Ttru_29 | Gulf of California | O | Perrin et al., 2011 |
| HQ206688.1 | CA_Ttru_30 | Gulf of California | U | Perrin et al., 2011 |
| HQ206689.1 | CA_Ttru_31 | Gulf of California | O | Perrin et al., 2011 |
| HQ206690.1 | CA_Ttru_32 | Gulf of California | U | Perrin et al., 2011 |
| HQ206691.1 | CA_Ttru_33 | Gulf of California | U | Perrin et al., 2011 |

**Continue…**

| **Accession number** | **Haplotype** | **Geographical location** | **E** | **Reference** |
| --- | --- | --- | --- | --- |
| **Pacific Ocean basin** | | | | |
| HQ206692.1 | CA_Ttru_34 | Gulf of California | U | Perrin et al., 2011 |
| HQ206693.1 | CA_Ttru_35 | Gulf of California | U | Perrin et al., 2011 |
| HQ206695.1 | CA_Ttru_37 | Gulf of California | U | Perrin et al., 2011 |
| HQ206696.1 | CA_Ttru_38 | Gulf of California | C | Perrin et al., 2011 |
| HQ206697.1 | CA_Ttru_39 | Gulf of California | O | Perrin et al., 2011 |
| HQ206698.1 | CA_Ttru_40 | Gulf of California | U | Perrin et al., 2011 |
| HQ206699.1 | CA_Ttru_41 | Gulf of California | O | Perrin et al., 2011 |
| HQ206700.1 | CA_Ttru_42 | Gulf of California | U | Perrin et al., 2011 |
| HQ206701.1 | CA_Ttru_43 | Gulf of California | U | Perrin et al., 2011 |
| HQ206703.1 | CA_Ttru_45 | Gulf of California | O | Perrin et al., 2011 |
| HQ206704.1 | CA_Ttru_46 | Gulf of California | O | Perrin et al., 2011 |
| HQ206705.1 | CA_Ttru_47 | Gulf of California | O | Perrin et al., 2011 |
| HQ206706.1 | CA_Ttru_48 | Gulf of California | O | Perrin et al., 2011 |
| HQ206709.1 | CA_Ttru_51 | Gulf of California | O | Perrin et al., 2011 |
| HQ206710.1 | CA_Ttru_52 | Gulf of California | O | Perrin et al., 2011 |
| HQ206711.1 | CA_Ttru_53 | Gulf of California | O | Perrin et al., 2011 |
| HQ206712.1 | CA_Ttru_54 | Gulf of California | O | Perrin et al., 2011 |
| HQ206713.1 | CA_Ttru_55 | Gulf of California | U | Perrin et al., 2011 |
| HQ206714.1 | CA_Ttru_56 | Gulf of California | O | Perrin et al., 2011 |
| EF672700.1 | SWFSC_Z18535 | Hawaiian Islands | P | Martien & Robertson, unpublished |
| EF672701.1 | SWFSC_Z18949 | Hawaiian Islands | P | Martien & Robertson, unpublished |
| EF672702.1 | SWFSC_Z18958 | Hawaiian Islands | P | Martien & Robertson, unpublished |
| EF672703.1 | SWFSC_Z27402 | Hawaiian Islands | P | Martien & Robertson, unpublished |
| EF672704.1 | SWFSC_Z27419 | Hawaiian Islands | P | Martien & Robertson, unpublished |
| EF672705.1 | SWFSC_Z27446 | Hawaiian Islands | P | Martien & Robertson, unpublished |

**Continue…**

| **Accession number** | **Haplotype** | **Geographical location** | **E** | **Reference** |
| --- | --- | --- | --- | --- |
| **Pacific Ocean basin** | | | | |
| EF672707.1 | SWFSC_Z30426 | Hawaiian Islands | P | Martien & Robertson, unpublished |
| EF672708.1 | SWFSC_Z30495 | Hawaiian Islands | P | Martien & Robertson, unpublished |
| EF672709.1 | SWFSC_Z30499 | Hawaiian Islands | P | Martien & Robertson, unpublished |
| EF672710.1 | SWFSC_Z33773 | Hawaiian Islands | P | Martien & Robertson, unpublished |
| EF672711.1 | SWFSC_Z33864 | Hawaiian Islands | P | Martien & Robertson, unpublished |
| EF672712.1 | SWFSC_Z33871 | Hawaiian Islands | P | Martien & Robertson, unpublished |
| EF672713.1 | SWFSC_Z33947 | Hawaiian Islands | P | Martien & Robertson, unpublished |
| EF672714.1 | SWFSC_Z33950 | Hawaiian Islands | P | Martien & Robertson, unpublished |
| EF672715.1 | SWFSC_Z33986 | Hawaiian Islands | P | Martien & Robertson, unpublished |
| EF672717.1 | SWFSC_Z34023 | Hawaiian Islands | P | Martien & Robertson, unpublished |
| EF672718.1 | SWFSC_Z34024 | Hawaiian Islands | P | Martien & Robertson, unpublished |
| EF672719.1 | SWFSC_Z49066 | Hawaiian Islands | P | Martien & Robertson, unpublished |
| EF672720.1 | SWFSC_Z49084 | Hawaiian Islands | P | Martien & Robertson, unpublished |
| EF672721.1 | SWFSC_Z49086 | Hawaiian Islands | P | Martien & Robertson, unpublished |
| EF672722.1 | SWFSC_Z49088 | Hawaiian Islands | P | Martien & Robertson, unpublished |
| EF672723.1 | SWFSC_Z49089 | Hawaiian Islands | P | Martien & Robertson, unpublished |
| EF672724.1 | SWFSC_Z53753 | Hawaiian Islands | P | Martien & Robertson, unpublished |
| AF355582.1 | T9 | China | U | Ji et al., unpublished |
| AF355583.1 | T8 | China | U | Ji et al., unpublished |
| AF355584.1 | T4 | China | U | Ji et al., unpublished |
| AF355586.1 | T1 | China | U | Ji et al., unpublished |
| AF355587.1 | T2 | China | U | Ji et al., unpublished |
| AF459522.1 | Ttr_0230 | China | U | Ji et al., unpublished |
| AF459523.1 | Ttr_0232 | China | U | Ji et al., unpublished |
| AB303154.1 | JTt01 | Japan | U | Kita et al., 2013 |
| AB303155.1 | JTt02 | Japan | U | Kita et al., 2013 |

**Continue…**

| **Accession number** | **Haplotype** | **Geographical location** | **E** | **Reference** |
| --- | --- | --- | --- | --- |
| **Pacific Ocean basin** | | | | |
| AB303157.1 | JTt04 | Japan | U | Kita et al., 2013 |
| AB303158.1 | JTt05 | Japan | U | Kita et al., 2013 |
| AB303160.1 | JTt07 | Japan | U | Kita et al., 2013 |
| AB303161.1 | JTt08 | Japan | U | Kita et al., 2013 |
| AB303164.1 | JTt11 | Japan | U | Kita et al., 2013 |
| AB303165.1 | JTt12 | Japan | U | Kita et al., 2013 |
| AB303166.1 | JTt13 | Japan | U | Kita et al., 2013 |
| AB303167.1 | JTt14 | Japan | U | Kita et al., 2013 |
| AB303168.1 | JTt15 | Japan | U | Kita et al., 2013 |
| AB303169.1 | JTt16 | Japan | U | Kita et al., 2013 |
| AB303170.1 | JTt17 | Japan | U | Kita et al., 2013 |
| AB303171.1 | JTt18 | Japan | U | Kita et al., 2013 |
| AB303172.1 | JTt19 | Japan | U | Kita et al., 2013 |
| AB303173.1 | JTt20 | Japan | U | Kita et al., 2013 |
| EU121118.1 | CRTtruBoINZ08 | New Zealand | C | Caballero et al., 2008 |
| **Black Sea** | | | | |
| KF570325.1 | BSEA2 | Black Sea | C | Moura et al., 2013 |
| KF570326.1 | BSEA3 | Black Sea | C | Moura et al., 2013 |
| KF570327.1 | BSEA1 | Black Sea | C | Moura et al., 2013 |
| KF570328.1 | BSEA6 | Black Sea | C | Moura et al., 2013 |
| KF570329.1 | BSEA7 | Black Sea | C | Moura et al., 2013 |
| KF570330.1 | BSEA5 | Black Sea | C | Moura et al., 2013 |
| KF570332.1 | BSEA8 | Black Sea | C | Moura et al., 2013 |
| KF570333.1 | BSEA9 | Black Sea | C | Moura et al., 2013 |
| AY963589.1 | BS2 | Black Sea | C | Natoli et al., 2004 |
| AY963593.1 | BS11 | Black Sea | C | Natoli et al., 2004 |

**Continue…**

| **Accession number** | **Haplotype** | **Geographical location** | **E** | **Reference** |
| --- | --- | --- | --- | --- |
| **Mediterranean Sea** | | | | |
| KF570316.1 | EMED4 | Eastern Mediterranean | C | Moura et al., 2013 |
| KF570317.1 | EMED5 | Eastern Mediterranean | C | Moura et al., 2013 |
| KF570319.1 | EMED2 | Eastern Mediterranean | C | Moura et al., 2013 |
| KF570320.1 | EMED10 | Eastern Mediterranean | C | Moura et al., 2013 |
| KF570321.1 | EMED6 | Eastern Mediterranean | C | Moura et al., 2013 |
| KF570322.1 | EMED9 | Eastern Mediterranean | C | Moura et al., 2013 |
| KF570323.1 | EMED7 | Eastern Mediterranean | C | Moura et al., 2013 |
| KF570324.1 | EMED8 | Eastern Mediterranean | C | Moura et al., 2013 |
| AY963594.1 | TtOG2 | East Mediterranean | C | Natoli et al., 2004 |
| AY963595.1 | TURC1c | East Mediterranean | C | Natoli et al., 2004 |
| AY963596.1 | TUR1c | East Mediterranean | C | Natoli et al., 2004 |
| AY963598.1 | Tt2/97 | East Mediterranean | C | Natoli et al., 2004 |
| AY963601.1 | TRIMonk | East Mediterranean | C | Natoli et al., 2004 |
| AY963604.1 | TtFil | East Mediterranean | C | Natoli et al., 2004 |
| AY963605.1 | CL551 | West Mediterranean | C | Natoli et al., 2004 |
| AY963606.1 | AATt9 | West Mediterranean | C | Natoli et al., 2004 |
| AY963607.1 | AATt13 | West Mediterranean | C | Natoli et al., 2004 |
| AY963608.1 | AATt14 | West Mediterranean | C | Natoli et al., 2004 |
| AY963610.1 | AATt25 | West Mediterranean | C | Natoli et al., 2004 |
| AY963612.1 | AATt3 | West Mediterranean | C | Natoli et al., 2004 |
| AY963613.1 | AATt16 | West Mediterranean | C | Natoli et al., 2004 |
| AY963614.1 | AATt17 | West Mediterranean | C | Natoli et al., 2004 |
| AY963615.1 | AAT43a | West Mediterranean | C | Natoli et al., 2004 |
| AY963616.1 | AAT47 | West Mediterranean | C | Natoli et al., 2004 |
| **Atlantic Ocean basin** | | | | |
| KF570345.1 | SCO7 | Scotland | C | Moura et al., 2013 |
| KF570352.1 | SCO5 | Scotland | C | Moura et al., 2013 |

**Continue…**

| **Accession number** | **Haplotype** | **Geographical location** | **E** | **Reference** |
| --- | --- | --- | --- | --- |
| **Atlantic Ocean basin** | | | | |
| KF570370.1 | WNAC11 | Western North Atlantic | C | Moura et al., 2013 |
| KF570371.1 | WNAC13 | Western North Atlantic | C | Moura et al., 2013 |
| KF570372.1 | WNAC14 | Western North Atlantic | C | Moura et al., 2013 |
| KF570373.1 | WNAC16 | Western North Atlantic | C | Moura et al., 2013 |
| KF570375.1 | WNAC22 | Western North Atlantic | C | Moura et al., 2013 |
| KF570376.1 | WNAC23 | Western North Atlantic | C | Moura et al., 2013 |
| KF570377.1 | WNAC25 | Western North Atlantic | C | Moura et al., 2013 |
| KF570378.1 | WNAC8 | Western North Atlantic | C | Moura et al., 2013 |
| KF570379.1 | WNAP11 | Western North Atlantic | C | Moura et al., 2013 |
| KF570380.1 | WNAP12 | Western North Atlantic | C | Moura et al., 2013 |
| KF570381.1 | WNAP17 | Western North Atlantic | P | Moura et al., 2013 |
| KF570383.1 | WNAP21 | Western North Atlantic | P | Moura et al., 2013 |
| KF570384.1 | WNAP22 | Western North Atlantic | P | Moura et al., 2013 |
| KF570385.1 | WNAP26 | Western North Atlantic | P | Moura et al., 2013 |
| KF570386.1 | WNAP7 | Western North Atlantic | P | Moura et al., 2013 |
| KF570387.1 | WNAP8 | Western North Atlantic | P | Moura et al., 2013 |
| KF570388.1 | WNAP9 | Western North Atlantic | P | Moura et al., 2013 |
| KT601188.1 | ENAC1 | Eastern North Atlantic | C | Nykanen & Foote, unpublished |
| KT601189.1 | ENAC2 | Eastern North Atlantic | C | Nykanen & Foote, unpublished |
| KT601194.1 | ENAC7 | Eastern North Atlantic | C | Nykanen & Foote, unpublished |
| KT601196.1 | ENAC9 | Eastern North Atlantic | C | Nykanen & Foote, unpublished |
| KT601197.1 | ENAP1 | Eastern North Atlantic | P | Nykanen & Foote, unpublished |
| KT601198.1 | ENAP2 | Eastern North Atlantic | P | Nykanen & Foote, unpublished |
| KT601199.1 | ENAP3 | Eastern North Atlantic | P | Nykanen & Foote, unpublished |
| KT601201.1 | ENAP5 | Eastern North Atlantic | P | Nykanen & Foote, unpublished |
| KT601202.1 | ENAP6 | Eastern North Atlantic | P | Nykanen & Foote, unpublished |

**Continue…**

| **Accession number** | **Haplotype** | **Geographical location** | **E** | **Reference** |
| --- | --- | --- | --- | --- |
| **Atlantic Ocean basin** | | | | |
| KT601207.1 | ENAP11 | Eastern North Atlantic | P | Nykanen & Foote, unpublished |
| AY963617.1 | TTR8 | Eastern North Atlantic | C | Natoli et al., 2004 |
| AY963618.1 | AAT49 | Eastern North Atlantic | C | Natoli et al., 2004 |
| AY963620.1 | AAT54 | Eastern North Atlantic | C | Natoli et al., 2004 |
| AY963621.1 | TtAL5 | Eastern North Atlantic | C | Natoli et al., 2004 |
| AY963626.1 | TTR1 | Eastern North Atlantic | C | Natoli et al., 2004 |
| DQ525358.1 | TT083 | Azores | P | Quérouil et al., 2007 |
| DQ525360.1 | TT085 | Azores | P | Quérouil et al., 2007 |
| DQ525361.1 | TT086 | Azores | P | Quérouil et al., 2007 |
| DQ525367.1 | TTM004 | Madeira | P | Quérouil et al., 2007 |
| DQ525368.1 | TTM005 | Madeira | P | Quérouil et al., 2007 |
| DQ525369.1 | TTM006 | Madeira | P | Quérouil et al., 2007 |
| DQ525370.1 | TTM007 | Madeira | P | Quérouil et al., 2007 |
| DQ525372.1 | TTM009 | Madeira | P | Quérouil et al., 2007 |
| DQ525373.1 | TTM010 | Madeira | P | Quérouil et al., 2007 |
| DQ525375.1 | TTM013 | Madeira | P | Quérouil et al., 2007 |
| DQ525377.1 | TTM015 | Madeira | P | Quérouil et al., 2007 |
| DQ525378.1 | TTM016 | Madeira | P | Quérouil et al., 2007 |
| DQ525381.1 | TTM022 | Madeira | P | Quérouil et al., 2007 |
| DQ073641.1 | TT001 | Azores | P | Quérouil et al., 2007 |
| DQ073642.1 | TT002 | Azores | P | Quérouil et al., 2007 |
| DQ073646.1 | TT006 | Azores | P | Quérouil et al., 2007 |
| DQ073650.1 | TT010 | Azores | P | Quérouil et al., 2007 |
| DQ073652.1 | TT012 | Azores | P | Quérouil et al., 2007 |
| DQ073661.1 | TT021 | Azores | P | Quérouil et al., 2007 |
| DQ073663.1 | TT023 | Azores | P | Quérouil et al., 2007 |

**Continue…**

| **Accession number** | **Haplotype** | **Geographical location** | **E** | **Reference** |
| --- | --- | --- | --- | --- |
| **Atlantic Ocean basin** | | | | |
| DQ073681.1 | TT041 | Azores | P | Quérouil et al., 2007 |
| DQ073688.1 | TT048 | Azores | P | Quérouil et al., 2007 |
| DQ073693.1 | TT053 | Azores | P | Quérouil et al., 2007 |
| DQ073698.1 | TT058 | Azores | P | Quérouil et al., 2007 |
| DQ073699.1 | TT059 | Azores | P | Quérouil et al., 2007 |
| DQ073701.1 | TT061 | Azores | P | Quérouil et al., 2007 |
| DQ073705.1 | TT066 | Azores | P | Quérouil et al., 2007 |
| DQ073707.1 | TT069 | Azores | P | Quérouil et al., 2007 |
| DQ073709.1 | TT072 | Azores | P | Quérouil et al., 2007 |
| DQ073710.1 | TT073 | Azores | P | Quérouil et al., 2007 |
| KX833116.1 | TTruCAR-BOC | Panama | P | Barragán-Barrera et al., 2017 |
| AF378176.1 | Haplotype B | Bahamas | C | Parson et al., unpublished |
| AF378177.1 | Haplotype D | Bahamas | C | Parson et al., unpublished |
| AF378178.1 | Haplotype E | Bahamas | C | Parson et al., unpublished |
| DQ118180.1 | Haplotype F | Bahamas | C | Parson et al., 2006 |
| DQ118181.1 | Haplotype G | Bahamas | C | Parson et al., 2006 |
| DQ118182.1 | Haplotype K | Bahamas | C | Parson et al., 2006 |
| DQ118183.1 | Haplotype L | Bahamas | C | Parson et al., 2006 |
| DQ118184.1 | Haplotype M | Bahamas | C | Parson et al., 2006 |
| AF155160.1 | - | Bahamas | C | Parson et al., 1999 |
| AF155161.1 | - | Bahamas | C | Parson et al., 1999 |
| AF155162.1 | - | Bahamas | C | Parson et al., 1999 |
| EF092943.1 | - | Texas | U | Harlin-Cognato & Honeycutt 2006 |
| NC_012059 | - | China | U | Xiong et al., 2009 |
| JF339982.1 | *Steno brenadensis* | Mexico | - | Vilstrup et al., 2011 |

**References**

**Barragán-Barrera DC, May-Collado LJ, Tezanos-Pinto G, Islas-Villanueva V, Correa-Cardenás CA, Caballero S. 2017.** High diversity structure and low mitochondrial diversity in bottlenose dolphins of the Archipelago of Bocas del Toro, Panama: a population at risk? *PLoS ONE* **12(12)**:e0189370. DOI: 10.1371/journal.pone.0189370.

**Caballero S, Jackson J, Mignucci-Giannoni AA, Barrios-Garrido H, Beltrán-Pedreros S, Robertson KM, Baker CS. 2008.** Molecular systematics of South American dolphins *Sotalia*: sister taxa determination and phylogenetic relationships, with insights into a multi-locus phylogeny of the Delphinidae. *Molecular Phylogenetic and Evolution* **46(1)**:252–268. DOI: 10.1016/j.ympev.2007.10.015.

**Harlin-Cognato AD, Honeycutt RL. 2006.** Multi-locus phylogeny of dolphins in the subfamily Lissodelphininae: character synergy improves phylogenetic resolution. *BMC Evolutionary Biology* **6(1)**:87. DOI: 10.1186/1471-2148-6-87.

**Ji G, Yang G, Liu S, Shou K.** A study on the variability of the mitochondrial DNA control region of bottlenose dolphins (genus: *Tursiops*) in Chinese waters. Unpublished.

**Kita YF, Hosomichi K, Suzuki S, Inoko H, Shiina T, Watanabe M, Tanaka A, Horie T, Ohizumi H, Tanaka S, Iwasaki T, Ota M, Kulski J. 2013.** Genetic and family structure in a group of 165 common bottlenose dolphins caught off the Japanese coast. *Marine Mammal Science* **29(3)**:474–496. DOI: 10.1111/j.1748-7692.2012.00581.x.

**Nykanen M. & Foote A.D.** Reconstructing the post-glacial colonization of the northern extreme of the range of a top marine predator, the bottlenose dolphin. Unpublished.

**Martien K, Robertson K.** Population structure of island-associated dolphins II: using mitochondrial and microsatellite markers of common bottlenose dolphins around the main Hawaiian Islands. Unpublished.

**Moura AE, Nielsen SC, Vilstrup JT, Moreno-Mayar JV, Gilbert MT, Gray HW, Natoli, A, Möller, L, Hoelzel AR*.* 2013.** Recent diversification of a marine genus (*Tursiops* spp.) tracks habitat preference and environmental change. *Systematic Biology* **62(6)**:865–877. DOI: 10.1093/sysbio/syt051.

**Natoli A, Peddemors VM, Hoelzel AR. 2004.** Population structure and speciation in the genus *Tursiops* based on microsatellite and mitochondrial DNA analyses. *Journal of Evolutionary Biology* **17(2)**:363–375. DOI: 10.1046/j.1420-9101.2003.00672.x.

**Parsons KM, Durban JW, Claridge DE, Balcomb Iii KC, Thompson PM, Noble LR.** Population genetics and social organization of bottlenose dolphins in the NE Bahamas. Unpublished.

**Parsons KM, Dallas JF, Claridge DE, Durban JW, Balcomb Iii KC, Thompson PM, Noble LR. 1999**. Amplifying dolphin mitochondrial DNA from faecal plumes. *Molecular Ecology* **8(10)**:1766–1768. DOI: 10.1046/j.1365x.1999.00723-8.x

**Parson KM, Durban JW, Claridge DE, Herzing DL, Balcomb KC, Noble LR. 2006.** Population genetic structure of coastal bottlenose dolphins (*Tursiops truncatus*) in the northern Bahamas. *Marine Mammal Science* **22(2)**:276–298. DOI: 10.1111/j.1748-7692.2006.00019.x.

**Perrin WF, Thieleking JL, Walter WA, Archer FI, Robertson KM. 2011.** Common bottlenose dolphins (*Tursiops truncatus*) in California waters: cranial differentiation of coastal and offshore ecotypes. *Marine Mammal Science* **27(4)**:769–792. DOI: 10.1111/j.1748-7692.2010.00442.x.

**Quérouil S, Silva MA, Freitas L, Prieto R, Magalhães S, Dinis A, Alves F, Matos JA, Mendoça D, Hammond PS, Santos RS*.* 2007.** High gene flow in oceanic bottlenose dolphins (*Tursiops truncatus*) of the North Atlantic. *Conservation Genetics* **8(6)**:1405–1419. DOI: 10.1007/s10592-007-9291-5.

**Segura I, Rocha-Olivares A, Flores-Ramirez S, Rojas-Bracho L. 2006.** Conservation implications of the genetic and ecological distinction of *Tursiops* *truncatus* ecotypes in the Gulf of California. *Biological Conservation* **133(3):**336–346. DOI: 10.1016/j.biocon.2006.06.017.

**Segura I, Rocha-Olivares A, Heckel G, Rojo-Arreola L, Gallo-Reynoso J, Hoelzel R.** Ecological affinity and restricted gene flow of the bottlenose dolphin, *Tursiops* *truncatus*, in the Gulf of California and Pacific Ocean. Unpublished.

**Vilstrup JT, Ho SY, Foote AD, Morin PA, Kreb D, Krützen M, Parra GJ, Robertson KM, de Stephanis R, Verborgh P, Willerslev E, Orlando L, Gilbert MTP. 2011.** Mitogenomic phylogenetic analyses of the Delphinidae with an emphasis on the Globicephalinae. *BMC Evolutionary Biology* **11**. DOI: 10.1186/1471-2148-1165.

**Xiong Y, Brandley MC, Xu S, Zhou K, Yang G. 2009.** Seven new dolphin mitochondrial genomes and a time-calibrated phylogeny of whales. *BMC* *Evolutionary Biology* **9(1)**:1–13. DOI: 10.1186/1471-2148-9-20.
