# Supplementary material for: Genetic divergence and fine scale population structure of the common bottlenose dolphin (Tursiops truncatus, Montagu) found in the Gulf of Guayaquil, Ecuador
Source: PeerJ. 2018 Apr 9;6:e4589. doi: 10.7717/peerj.4589 (PMC5916226; doi:10.7717/peerj.4589)
Supplement: Supplemental Information 7 — The table includes the accession numbers, sequences geographic location, ecotypes, and the species. Acronyms: C: coastal, P: pelagic, U: unknown. [file peerj-06-4589-s007.docx]

| **Species** | **Geographic location** | **Ecotype** | **Accession number** | **References** |
| --- | --- | --- | --- | --- |
| *Tursiops truncatus* | China | U | NC_012059.1 | Xiong et al., 2009 |
| *Tursiops truncatus* | Gulf of California | C | KF570389.1 | Moura et al., 2013 |
| *Tursiops truncatus* | Western North Atlantic | C | KF570370.1- KF570378.1 | Moura et al., 2013 |
| *Tursiops truncatus* | Western North Atlantic | P | KF570379.1- KF570388.1 | Moura et al., 2013 |
| *Tursiops truncatus* | Eastern North Atlantic | C | KT601188.1- KT601190.1 | Nykanen & Foote, unpublished |
| *Tursiops truncatus* | Eastern North Atlantic | C | KT601192.1 | Nykanen & Foote, unpublished |
| *Tursiops truncatus* | Eastern North Atlantic | C | KT601194.1- KT601196.1 | Nykanen & Foote, unpublished |
| *Tursiops truncatus* | Eastern North Atlantic | P | KT601197.1- KT601203.1 | Nykanen & Foote, unpublished |
| *Tursiops truncatus* | Eastern North Atlantic | P | KT601206.1 | Nykanen & Foote, unpublished |
| *Tursiops truncatus* | Eastern North Atlantic | P | KT601206.1 | Nykanen & Foote, unpublished |
| *Tursiops truncatus* | Scotland | C | KF570345.1 | Moura et al., 2013 |
| *Tursiops truncatus* | Scotland | C | KF570346.1 | Moura et al., 2013 |
| *Tursiops truncatus* | Scotland | C | KF570347.1 | Moura et al., 2013 |
| *Tursiops truncatus* | Scotland | C | KF570351.1 | Moura et al., 2013 |
| *Tursiops truncatus* | Scotland | C | KF570352.1 | Moura et al., 2013 |
| *Tursiops truncatus* | Black Sea | C | KF570325.1- KF570330.1 | Moura et al., 2013 |
| *T. truncatus ponticus* | Black Sea | C | KF570332.1- KF570334.1 | Moura et al., 2013 |
| *Tursiops truncatus* | Eastern Mediterranean | C | KF570315.1- KF570323.1 | Moura et al., 2013 |
| *Tursiops aduncus* | China | U | NC_012058.1 | Xiong et al., 2009 |
| *Tursiops aduncus* | Eastern Australia | C | KF570335.1 | Moura et al., 2013 |
| *Tursiops aduncus* | Eastern Australia | C | KF570337.1 | Moura et al., 2013 |
| *Tursiops aduncus* | South Africa | C | KF570362.1 | Moura et al., 2013 |
| *“ Tursiops australis”* | Southern Australia | C | NC_022805.1 | Moura et al., 2013 |
| *“ Tursiops australis”* | Southern Australia | C | KF570365.1 | Moura et al., 2013 |
| *Steno brenadensis* | Mexico | - | JF339982.1 | Vilstrup et al., 2011 |

**References**

**Nykanen M. & Foote A.D.** Reconstructing the post-glacial colonisation of the northern extreme of the range of a top marine predator, the bottlenose dolphin. Unpublished.

**Moura AE, Nielsen SC, Vilstrup JT, Moreno-Mayar JV, Gilbert MT, Gray HW, Natoli, A, Möller, L, Hoelzel AR*.* 2013.** Recent diversification of a marine genus (*Tursiops* spp.) tracks habitat preference and environmental change. *Systematic Biology* **62(6)**:865–877. DOI: 10.1093/sysbio/syt051.

**Vilstrup JT, Ho SY, Foote AD, Morin PA, Kreb D, Krützen M, Parra GJ, Robertson KM, de Stephanis R, Verborgh P, Willerslev E, Orlando L, Gilbert MTP. 2011.** Mitogenomic phylogenetic analyses of the Delphinidae with an emphasis on the Globicephalinae. *BMC Evolutionary Biology* **11**. DOI: 10.1186/1471-2148-1165.

**Xiong Y, Brandley MC, Xu S, Zhou K, Yang G. 2009.** Seven new dolphin mitochondrial genomes and a time-calibrated phylogeny of whales. *BMC* *Evolutionary Biology* **9(1)**:1–13. DOI: 10.1186/1471-2148-9-20.
