# Supplementary material for: Genetic divergence and fine scale population structure of the common bottlenose dolphin (Tursiops truncatus, Montagu) found in the Gulf of Guayaquil, Ecuador
Source: PeerJ. 2018 Apr 9;6:e4589. doi: 10.7717/peerj.4589 (PMC5916226; doi:10.7717/peerj.4589)
Supplement: Supplemental Information 8 — Number of samples by haplotype (n). Acronyms: Hap: Haplotype, Ttr: Tursiops truncatus, * samples excluded from genetic diversity, population structure, and genetic divergence analyses. [file peerj-06-4589-s008.docx]

| **Code** | **n** | **Sample ID** | **Sampling location** | **Accession number** |
| --- | --- | --- | --- | --- |
| Hap 1 | 1 | Ttr_2 | Salinas, Punta Carnero | KU992129 |
| Hap 2 | 2 | Ttr_3, Ttr_8 | Salinas, Mar Bravo | KU992130, KU992135 |
| Hap 3 | 1 | Ttr_4 | Salinas, Mar Bravo | KU992131 |
| Hap 4 | 2 | Ttr_5*,* Ttr_37 | Salinas, Mar Bravo | KU992132, KU992121 |
| Hap 5 | 1 | Ttr_6 | Salinas, Mar Bravo | KU992133 |
| Hap 6 | 1 | Ttr_7 | Salinas, Mar Bravo | KU992134 |
| Hap 7 | 27 | Ttr_10, Ttr_33 | Jambelí Island | KU992120, KU992138 |
|  |  | Ttr_17 | General Villamil, Playas | KU992121 |
|  |  | Ttr_21, Ttr_23 | Puná Island | KU992122, KU992124 |
|  |  | Ttr_38-Ttr_41, Ttr_43-Ttr_45, Ttr_49, Ttr_52, Ttr_56, Ttr_57, Ttr_63, Ttr_64 Ttr_68 | Posorja | KU992141-KU992144 KU992146-KU992148 KU992152, KU992155 KU992159, KU992160 KU992166, KU992167 KU992171 |
|  |  | Ttr_48*,*  Ttr_58- Ttr_62 | El Morro | KU992151, KU992161-KU992165 |
|  |  | Ttr_26, Ttr_28 | Unknown* | KU992125, KU992126 |
| Hap 8 | 2 | Ttr_16 | Puná Island | KU992136 |
|  |  | Ttr_22 | Unknown* | KU992123 |
| Hap 9 | 5 | Ttr_20 | Puná Island | KU992137 |
|  |  | Ttr_46, Ttr_55 Ttr_66, Ttr_67 | Posorja Harbor | KU992149, KU992158 KU992169, KU992170 |
| Hap 10 | 2 | Ttr_1 | Galápagos Islands* | KU992128 |
|  |  | Ttr_35 | Salinas, Mar Bravo | KU992139 |
| Hap 11 | 1 | Ttr_36 | Salinas, Mar Bravo | KU992140 |
| Hap 12 | 3 | Ttr_42, Ttr_50 Ttr_51 | Posorja | KU992145, KU992153 KU992154 |
| Hap 13 | 4 | Ttr_47 | El Morro | KU992150 |
|  |  | Ttr_53, Ttr_54 | Puná Island | KU992156, KU992157 |
|  |  | Ttr_65 | Posorja | KU992168 |
| Hap 14 | 1 | Ttr_69 | Peru: Santa Rosa* | KU992172 |
