# Supplementary material for: Genetic divergence and fine scale population structure of the common bottlenose dolphin (Tursiops truncatus, Montagu) found in the Gulf of Guayaquil, Ecuador
Source: PeerJ. 2018 Apr 9;6:e4589. doi: 10.7717/peerj.4589 (PMC5916226; doi:10.7717/peerj.4589)
Supplement: Supplemental Information 9 — The table includes the accession numbers of the sequences which are similar to the sequences found in the present study. 100% of similarity is in bold. 95% of similarity is in red. Acronyms: H#: number of haplotype; Hap: Haplotype, Ttr: Tursiops truncatus, CR: Control region, COI: cytochrome oxidase I, COII: cytochrome oxidase II, ND1-ND2: NADH dehydrogenase subunit I-II, Cyt b: cytochrome b. [file peerj-06-4589-s009.docx]

| **Sample ID** | **1,050 bp 12S-16S** | | **562 bp 16S rRNA** | | **897 bp *ND1*-*ND2*** | | **837 bp *COI*** | | **738 bp *COII*** | | **436 bp *Cytb*** | | **715 bp CR** | | **Concatenated** |
| --- | --- | --- | --- | --- | --- | --- | --- | --- | --- | --- | --- | --- | --- | --- | --- |
|  | **H#** | **Identity** | **H#** | **Identity** | **H#** | **Identity** | **H#** | **Identity** | **H#** | **Identity** | **H#** | **Identity** | **H#** | **Identity** | **Haplotype** |
| Ttr_1 | H1 | KF570386 | H1 | KF570386 | H1 | KF570386 | H1 | KF570386 | H1 | KF570386 | H1 | KF570389 | H1 | KT601198 | Hap 27 |
| Ttr_2 | H2 | KF570389 | H2 | KF570389 | H2 | KF570389 | H2 | **KF312622** | H2 | KF570389 | H2 | **KF570386** | H2 | KF570389 | Hap 1 |
| Ttr_3 | H3 | KF570389 | H3 | KF570389 | H3 | KF570389 | H3 | KF570383 | H3 | KF570386 | H3 | KF570386 | H3 | HE680129 | Hap 2 |
| Ttr_4 | H4 | KF570389 | H1 | KF570386 | H4 | KF570389 | H4 | KF570315 | H4 | KF570383 | H4 | KF570386 | H4 | KF570389 | Hap 3 |
| Ttr_5 | H5 | **KF570389** | H1 | KF570386 | H5 | KF570389 | H5 | KF312622 | H5 | KF570389 | H2 | **KF570386** | H5 | KF570389 | Hap 4 |
| Ttr_6 | H1 | KF570386 | H1 | KF570386 | H1 | KF570386 | H1 | KF570386 | H1 | KF570386 | H1 | KF570389 | H6 | DQ073656 | Hap 5 |
| Ttr_7 | H5 | **KF570389** | H1 | KF570386 | H6 | KF570389 | H6 | KF312622 | H6 | KF570386 | H5 | KF570386 | H7 | KF570389 | Hap 6 |
| Ttr_8 | H3 | KF570389 | H3 | KF570389 | H3 | KF570389 | H3 | KF570383 | H3 | KF570386 | H3 | KF570386 | H3 | HE680129 | Hap 2 |
| Ttr_38 | H3 | KF570389 | H3 | KF570389 | H3 | KF570389 | H7 | DQ466015 | H3 | KF570386 | H3 | KF570386 | H8 | DQ073666 | Hap 7 |
| Ttr_39 | H3 | KF570389 | H3 | KF570389 | H3 | KF570389 | H8 | DQ466015 | H3 | KF570386 | H3 | KF570386 | H8 | DQ073666 | Hap 8 |
| Ttr_40 | H3 | KF570389 | H3 | KF570389 | H3 | KF570389 | H8 | DQ466015 | H3 | KF570386 | H3 | KF570386 | H8 | DQ073666 | Hap 8 |
| Ttr_41 | H3 | KF570389 | H3 | KF570389 | H3 | KF570389 | H8 | DQ466015 | H3 | KF570386 | H3 | KF570386 | H8 | DQ073666 | Hap 8 |
| Ttr_42 | H3 | KF570389 | H1 | KF570386 | H3 | KF570389 | H8 | DQ466015 | H3 | KF570386 | H3 | KF570386 | H9 | HE680099 | Hap 9 |
| Ttr_43 | H3 | KF570389 | H1 | KF570386 | H3 | KF570389 | H9 | DQ466015 | H3 | KF570386 | H3 | KF570386 | H10 | DQ073666 | Hap 10 |
| Ttr_44 | H3 | KF570389 | H3 | KF570389 | H3 | KF570389 | H10 | DQ466015 | H3 | KF570386 | H3 | KF570386 | H8 | DQ073666 | Hap 11 |
| Ttr_45 | H3 | KF570389 | H3 | KF570389 | H3 | KF570389 | H11 | KC312621 | H3 | KF570386 | H6 | KF570386 | H8 | DQ073666 | Hap 12 |
| Ttr_46 | H3 | KF570389 | H1 | KF570386 | H3 | KF570389 | H9 | DQ466015 | H3 | KF570386 | H3 | KF570386 | H11 | HE680099 | Hap 13 |
| Ttr_47 | H3 | KF570389 | H3 | KF570389 | H3 | KF570389 | H12 | KC312621 | H3 | KF570386 | H3 | KF570386 | H12 | DQ073666 | Hap 14 |
| Ttr_48 | H3 | KF570389 | H3 | KF570389 | H3 | KF570389 | H7 | DQ466015 | H3 | KF570386 | H3 | KF570386 | H8 | DQ073666 | Hap 7 |
| Ttr_49 | H3 | KF570389 | H3 | KF570389 | H3 | KF570389 | H13 | KX857349 | H3 | KF570386 | H3 | KF570386 | H13 | DQ073666 | Hap 15 |
| Ttr_50 | H3 | KF570389 | H1 | KF570386 | H3 | KF570389 | H14 | KF570383 | H3 | KF570386 | H3 | KF570386 | H9 | HE680099 | Hap 16 |
| Ttr_51 | H3 | KF570389 | H1 | KF570386 | H3 | KF570389 | H14 | KF570383 | H3 | KF570386 | H3 | KF570386 | H9 | HE680099 | Hap 16 |
| Ttr_52 | H3 | KF570389 | H3 | KF570389 | H3 | KF570389 | H14 | KF570383 | H3 | KF570386 | H6 | KF570386 | H8 | DQ073666 | Hap 17 |
| Ttr_53 | H3 | KF570389 | H1 | KF570386 | H3 | KF570389 | H8 | DQ466015 | H3 | KF570386 | H3 | KF570386 | H14 | DQ073666 | Hap 18 |
| Ttr_54 | H3 | KF570389 | H1 | KF570386 | H3 | KF570389 | H8 | DQ466015 | H3 | KF570386 | H3 | KF570386 | H14 | DQ073666 | Hap 18 |
| Ttr_55 | H3 | KF570389 | H1 | KF570386 | H3 | KF570389 | H15 | DQ466015 | H3 | KF570386 | H3 | KF570386 | H11 | HE680099 | Hap 19 |
| Ttr_56 | H3 | KF570389 | H3 | KF570389 | H3 | KF570389 | H15 | DQ466015 | H3 | KF570386 | H6 | KF570386 | H 8 | DQ073666 | Hap 20 |

Continue…

| **Sample ID** | **1,050 bp 12S-16S** | | **562 bp 16S rRNA** | | **897 bp *ND1*-*ND2*** | | **837 bp *COI*** | | **738 bp *COII*** | | **436 bp *Cytb*** | | **715 bp CR** | | **Concatenated** |
| --- | --- | --- | --- | --- | --- | --- | --- | --- | --- | --- | --- | --- | --- | --- | --- |
|  | **H#** | **Identity** | **H#** | **Identity** | **H#** | **Identity** | **H#** | **Identity** | **H#** | **Identity** | **H#** | **Identity** | **H#** | **Identity** | **Haplotype** |
| Ttr_57 | H3 | KF570389 | H3 | KF570389 | H3 | KF570389 | H8 | DQ466015 | H3 | KF570386 | H3 | KF570386 | H8 | DQ073666 | Hap 8 |
| Ttr_58 | H3 | KF570389 | H3 | KF570389 | H3 | KF570389 | H8 | DQ466015 | H3 | KF570386 | H3 | KF570386 | H8 | DQ073666 | Hap 8 |
| Ttr_59 | H3 | KF570389 | H3 | KF570389 | H3 | KF570389 | H8 | DQ466015 | H3 | KF570386 | H3 | KF570386 | H8 | DQ073666 | Hap 8 |
| Ttr_60 | H3 | KF570389 | H1 | KF570386 | H3 | KF570389 | H8 | DQ466015 | H3 | KF570386 | H3 | KF570386 | H8 | DQ073666 | Hap 21 |
| Ttr_61 | H3 | KF570389 | H3 | KF570389 | H3 | KF570389 | H8 | DQ466015 | H3 | KF570386 | H3 | KF570386 | H10 | DQ073666 | Hap 22 |
| Ttr_62 | H3 | KF570389 | H3 | KF570389 | H3 | KF570389 | H15 | DQ466015 | H3 | KF570386 | H3 | KF570386 | H8 | DQ073666 | Hap 23 |
| Ttr_63 | H3 | KF570389 | H3 | KF570389 | H3 | KF570389 | H8 | DQ466015 | H3 | KF570386 | H3 | KF570386 | H8 | DQ073666 | Hap 8 |
| Ttr_64 | H3 | KF570389 | H3 | KF570389 | H3 | KF570389 | H15 | DQ466015 | H3 | KF570386 | H3 | KF570386 | H8 | DQ073666 | Hap 23 |
| Ttr_65 | H3 | KF570389 | H3 | KF570389 | H3 | KF570389 | H8 | DQ466015 | H3 | KF570386 | H3 | KF570386 | H12 | DQ073666 | Hap 24 |
| Ttr_66 | H3 | KF570389 | H1 | KF570386 | H3 | KF570389 | H8 | DQ466015 | H3 | KF570386 | H3 | KF570386 | H11 | HE680099 | Hap 25 |
| Ttr_67 | H3 | KF570389 | H1 | KF570386 | H3 | KF570389 | H15 | DQ466015 | H3 | KF570386 | H3 | KF570386 | H11 | HE680099 | Hap 19 |
| Ttr_68 | H3 | KF570389 | H1 | KF570386 | H3 | KF570389 | H8 | DQ466015 | H3 | KF570386 | H3 | KF570386 | H10 | DQ073666 | Hap 26 |
| Ttr_69 | H3 | KF570389 | H1 | KF570386 | H3 | KF570389 | H16 | KF570383 | H3 | KF570386 | H3 | KF570386 | H15 | DQ073643 | Hap 28 |
