# Supplementary material for: Genetic divergence and fine scale population structure of the common bottlenose dolphin (Tursiops truncatus, Montagu) found in the Gulf of Guayaquil, Ecuador
Source: PeerJ. 2018 Apr 9;6:e4589. doi: 10.7717/peerj.4589 (PMC5916226; doi:10.7717/peerj.4589)
Supplement: Supplemental Information 10 — Total number of sequences (n), base pair (bp), polymorphic sites (S), non-coding (12S rRNA, 16S rRNA, and tRNAs), Protein-coding genes (ND1-ND2, COI, COII, and Cyt b). [file peerj-06-4589-s010.docx]

| **Mitochondrial region** | **n** | **Model** | **Length (bp)** | **S** | **Parsimony informative sites** | **Gaps** |
| --- | --- | --- | --- | --- | --- | --- |
| Non-coding | 94 | TPM1uf+I | 1,896 | 96 | 48 | 1 |
| Control region | 94 | TPM3uf+I+G | 698 | 91 | 64 | 16 |
| Protein-coding genes | 94 | TVM+I+G | 2,615 | 363 | 233 | 1 |
| Total | 94 |  | 5,209 | 550 | 345 | 18 |
